# Uncovering the Research Gaps to Alleviate the Negative Impacts of Climate Change on Food Security: A Review

**DOI:** 10.3389/fpls.2022.927535

**Published:** 2022-07-11

**Authors:** Muhammad Shahbaz Farooq, Muhammad Uzair, Ali Raza, Madiha Habib, Yinlong Xu, Muhammad Yousuf, Seung Hwan Yang, Muhammad Ramzan Khan

**Affiliations:** ^1^Institute of Environment and Sustainable Development in Agriculture, Chinese Academy of Agricultural Sciences (CAAS), Beijing, China; ^2^National Institute for Genomics and Advanced Biotechnology, Islamabad, Pakistan; ^3^College of Agriculture, Oil Crops Research Institute, Fujian Agriculture and Forestry University (FAFU), Fuzhou, China; ^4^Pakistan Agricultural Research Council, Islamabad, Pakistan; ^5^Department of Biotechnology, Chonnam National University, Yeosu, South Korea

**Keywords:** climate-smart food, environmental stresses, food safety, future crops, implementation challenges, climate adaptation

## Abstract

Climatic variability has been acquiring an extensive consideration due to its widespread ability to impact food production and livelihoods. Climate change has the potential to intersperse global approaches in alleviating hunger and undernutrition. It is hypothesized that climate shifts bring substantial negative impacts on food production systems, thereby intimidating food security. Vast developments have been made addressing the global climate change, undernourishment, and hunger for the last few decades, partly due to the increase in food productivity through augmented agricultural managements. However, the growing population has increased the demand for food, putting pressure on food systems. Moreover, the potential climate change impacts are still unclear more obviously at the regional scales. Climate change is expected to boost food insecurity challenges in areas already vulnerable to climate change. Human-induced climate change is expected to impact food quality, quantity, and potentiality to dispense it equitably. Global capabilities to ascertain the food security and nutritional reasonableness facing expeditious shifts in biophysical conditions are likely to be the main factors determining the level of global disease incidence. It can be apprehended that all food security components (mainly food access and utilization) likely be under indirect effect via pledged impacts on ménage, incomes, and damages to health. The corroboration supports the dire need for huge focused investments in mitigation and adaptation measures to have sustainable, climate-smart, eco-friendly, and climate stress resilient food production systems. In this paper, we discussed the foremost pathways of how climate change impacts our food production systems as well as the social, and economic factors that in the mastery of unbiased food distribution. Likewise, we analyze the research gaps and biases about climate change and food security. Climate change is often responsible for food insecurity issues, not focusing on the fact that food production systems have magnified the climate change process. Provided the critical threats to food security, the focus needs to be shifted to an implementation oriented-agenda to potentially cope with current challenges. Therefore, this review seeks to have a more unprejudiced view and thus interpret the fusion association between climate change and food security by imperatively scrutinizing all factors.

## General Background and Climate Change Status

Negative impacts of climate change on global food security components is one of the major threats in the current century and their alleviation is essential to fulfill the future food demands of increasingly inflating population. Climate change has already threatened crop productivity especially in the major food crops (wheat, maize, rice) which are staple food crops across many countries (Lobell et al., 2011; Syed et al., 2022). The impacts of climate change on major food crops have been considered comprehensively, but the impacts on livestock and fisheries which are also important in food security have been ignored (Herrero et al., 2015; Campbell et al., 2016). Climate variables like low and high temperature stresses, change in precipitation frequency and intensity, and other climate change-induced disasters such as drought, salinity and erupting sea levels are changing slowly, but definitely will negatively impact the crop production in the coming decades (Raza et al., 2021; Ullah et al., 2021). Therefore, farmers will have to deal with climate change and extreme climate events that consequently will make the agricultural farming more difficult (IPCC, 2012). Therefore, climate mitigation and adaptation approaches must be adopted before major crisis happens. Certainly, there are many research limitations concerning research conduction and research implementation regarding climate change and impacts on food production and food security (Knight et al., 2008; Firdaus et al., 2019). Meanwhile, some uncertainties are associated with climate; not sure what fluctuations will come in climate and to what extent these changes will impacts food production. These uncertainties have been existing for decades and likely to remain hampering the food security goals in the coming decades unless the proper research-based strategies are undertaken (Heal and Millner, 2014a). Long-term decision-making is more challenging and hindered due to the uncertainties regarding climate change impacts on food security. Therefore, it is necessary to alleviate the research gaps between climate change and food security thereby undertaking mitigation and adaptation approaches to improve the decisions and actions for a climate-smart food production system ensuring food security.

It is univocal that human-induced activities have increased the warming process of the atmosphere, ocean and land. Widespread and rapid changes have occurred in the atmosphere, cryosphere, ocean, and biosphere. Human activities have substantially and unequivocally increased the well-mixed GHGs concentrations since 1750. Change analysis in concentration of GHGs since 2011 have depicted a continued increase in the atmosphere for major gases, approaching annual averages of 410 ppm for CO_2_, 1866 ppb for CH_4_, and 332 ppb for N_2_O till 2019 (Myhre et al., 2016). During last four decades, the warming process have been rapidly increased successively warmer than any of the decade that predated it since 1850s. Global mean surface temperature during 2001–2020 was higher by 0.99°C than 1850–1900. Moreover, global mean surface temperature was 1.09°C higher in 2011–2020 than 1850–1900, with rapid warming over earth surface (nearly 1.59°C) than over the ocean (nearly 0.88°C; IPCC, 2021). However, the projected increase in global mean surface temperature is principally due to further warming during 2003–2012 (+0.19°C). The relative projected extent of total human-induced global mean surface warming during 1850–1900 to 2010–2019 is varying between 0.8 and 1.3°C, with a more accurate estimate of 1.07°C. It is anticipated that mixture of different GHGs bestowed a warming of 1.0–2.0°C, other human factors shared a mean temperature change to 0.8°C, natural factors altered global mean surface temperature varied between –0.1 and + 0.1°C, while internal variabilities changed surface temperature ranged between –0.2 and + 0.2°C (Zhou, 2021). It has been projected that mixture of different GHGs could be the major contributor for tropospheric warming since 1979 and more obviously human-based activities caused stratospheric ozone depletion during 1979–1990s (Andersen et al., 2013).

Global projections for annual precipitation change over earth surface have depicted an increase since 1950s, with a rapid increase rate since the 1980s. It is presumptive that human activities have contributed to the patterns of observed precipitation shifts since the 20th century and more likely subjected toward near-surface ocean salinity (Hatfield and Prueger, 2015; IPCC, 2021). The tracks for mid-latitude storms have been shifted poleward in both hemispheres since the 1980s, with noticeable seasonality in change trends. Human-based activities are anticipated to be the major contributors of the global retreat of glaciers since the 1990s and also caused decrease in Arctic sea ice area during 1979–2019 (Wang and Zhou, 2019). No significant change trend has been observed in Antarctic Sea ice area during 1979–2020 due to regionally contending trends and enormous internal variabilities, whereas, decrease in Northern Hemisphere spring snow cover was seen after 1950s. Moreover, it is more probable that human influences have assisted to the observed surface melting of the Greenland Ice Sheet during recent years, however, there exists a limited evidence, with a uncertain agreement, of human-induced impacts on the Antarctic Ice losses (Moon et al., 2018; Aschwanden et al., 2019).

Atmospheric CO_2_ concentrations have become higher than at any time during last 2 million years, and concentrations of CH_4_ and N_2_O have become higher than at any time in last 0.8 million years. Alongside, human-induced climate change has already been affecting and changing the magnitude of climate extreme events across the globe. Indication of observed shifts in extreme events such as heatwaves, frequent and irregular precipitation, droughts, and tropical cyclones, and, specifically, the ascription of these extreme events to human influence, has also been strengthened (Castillo et al., 2021). It has been certainly observed that hot extreme events have become more intense and frequent across several global regions since 1950s, while cold waves have relatively become less severe and less frequent (Zheng et al., 2020), where human-caused climate change is one of the main drivers of these extreme events (Bell et al., 2018). During recent years, severe heatwaves have been observed that would have been seriously improbable to occur without human activities causing climate change. Marine heatwaves have nearly doubled in frequency since the 1980s due to irregular human-based activities which more likely boosted the heatwaves after 2000s (Oliver et al., 2019). Moreover, the frequency and intensity of severe precipitation events have shown an increasing trend after 1950s across the various regions worldwide due to many natural and human-caused factors. This irregular change in annual precipitation and occurrence of frequent heatwaves have led toward ecological drought events in various regions due to increased evapotranspiration (Jiménez-Donaire et al., 2020; Ayugi et al., 2022). Decline in global monsoon precipitations has been observed between 1950s and 1980s which could partially be attributed to human-induced Northern Hemisphere aerosol emissions. However, an increase in precipitation after 1980s have resulted from rising atmospheric GHGs levels and decadal to multi-decadal internal modulations (Johnson et al., 2020). Considering West Africa, East and South Asian regions, increases in annual monsoon precipitation caused by warming from mixed GHGs emissions were subverted by decline in annual monsoon precipitation due to cooling from human-induced aerosol emissions since the 20th century (Tian et al., 2018; Herbert et al., 2022). Whereas, an increase in West African monsoon precipitation since the 1980s was observed might be due to the growing influence of severe GHGs emissions and decrease in the cooling effects of human-induced aerosol emissions over European and North American regions. In the same way, the occurrence of tropical cyclones has increased over the recent decades, and it has been projected that the latitude where tropical cyclones in the Western- Northern-Pacific hit their extremum intensity has switched northward which happened due to several internal and external factors (Feng et al., 2021). There are many uncertainties in multi-decadal change trends in the frequency and intensity of categorical tropical cyclones. It has been indicated that human-caused climate change generally causes heavy precipitation events associated with tropical cyclones, however, limited data availability precludes clear identification of previous trends on the global scale. Irregular and uncontrolled human-based activities have increased the chance of co-occurrence of compound extreme events since 1950s which includes increase in the frequency of simultaneous heatwave and drought events globally, wildfires weather in various parts of all tenanted continents, and intensified flooding (Ye et al., 2019; Coscarelli et al., 2021).

Long before human existence and activities, the earth has gone through different fluctuations of cooling and warming in the past. The major natural factors that share to climate change include sun’s intensity, natural GHGs concentrations, volcanic eruptions, orbital changes, movement of crustal plates, El Nino-Southern Oscillation (ENSO; Thompson, 2010; Rosso Grossman, 2018; Santos and Bakhshoodeh, 2021). However, the projections and analyses have shown that earth warming primarily occurred since the last century is happening at rapid speed which cannot be interpretated only by natural causes. It has been explained there can be different cyclical shifts in earth’s orbit and tilt that subject toward the climate changes. Volcanic eruptions subject toward the discharge of CO_2_, along with emissions of different aerosols which include dust or volcanic ash, and sulfur dioxide (SO_2_; Reikard, 2019). Generally, aerosols may be solids or liquids that stream around in the atmosphere which may also include dust, soot, salt crystals, viruses, and bacteria. Aerosols disperse the ingress solar radiation, leading toward a mild cooling effect. Whereas, volcanic aerosols can even restrict a major percentage of solar radiations and may cause a cooling effect that may last for years (Piazzola et al., 2021). Heavy winds transfer the solid and liquid aerosols across the globe toward eastern or western regions. Hence, volcanoes that generally erupt at lower latitudes near to the equator are expected to subject toward hemispheric or worldwide cooling effects. Meanwhile, volcanoes that erupt near to the poles or higher latitudes are uncertain whether they will cause cooling because the sulfurous aerosols are restricted to wind patterns around the poles (Sun et al., 2019). The total amount of solar radiation touching the earth surface varies by little margins as the energy released by the sun only differs by 1.3 W/m^2^ which is associated with darker areas on the sun termed as sunspots (Hussain et al., 2018; Adnan et al., 2020). Approximately after every decade, the total number of darker areas on the sun changes from a maximum to a minimum number. The sun releases marginally more active radiation during active periods of darker spots. As the darker spots suppress the heat, that heat transports toward the surrounding areas causing these regions to be brighter than routine, and emitting more heat. Moreover, higher number of sunspots can share toward warmer climate, while less number appear to contribute toward a cooler global climate. As tectonic crustal plates shift across the geological timescales, landmasses are transported along to different places and latitudes which ultimately impact the global circulation patterns of seas and ocean waters, air movement, and the climate of the continents (Van Der Meer et al., 2014).

Climate change is considered as a universal truth across the globe with subsequent untoward impacts on agricultural food production, water resources, biodiversity, human livelihoods, forest farming systems, and socio-economic components (Muluneh, 2021). It has been projected that due to anticipated global climate change, developing and underdeveloped countries will suffer from adverse impacts because of less adaptive capacity. At the regional scale, the most vulnerable are the ordinary citizens especially the poor and insure the destructive reverberations of climate change owing to the scarcity of required resources, capacity building, and limited access to the information (Barnett et al., 2014). Climatic uncertainties have become more frequent and intense, due to irregular anthropogenic activities that subject toward climate change which continue to intensify the ecological disasters (Mahmoud and Gan, 2018; Trenberth, 2018). At societal level, the poorest communities become the principal victims of these ecological disasters with poor access to capacity building and lowest dawdling incomes without being the major sharer to climate change. Uncontrolled urbanization and industrial revolution have increased the greenhouse gases (GHGs) emissions which massively caused the intensified global warming with prolonged capacity (even for decades) to render the warming process. The probable challenges of undernourishment, malnutrition, and food insecurity are the most significant impacts of climate change. The irregular climatic patterns likely to have unfavorable impacts on livelihoods, household incomes, and food security since climate extreme events ruin essential infrastructures, agricultural systems, and other public properties, and subsequently increase poverty. Uncertain and irregular climate changes are causing the incidence of ecological disasters which further impact the livelihoods. The climate change associated extreme events include faster glacial thawing, sea level rise, drought, floods and salinity which will adversely impact the livelihoods and social emotions (Iniguez-Gallardo et al., 2021), land-use, dependability and amount of available irrigational water and other agricultural resources (Davidson, 2018).

### Global Climate Change and Impacts on Food Security

Concrete reports on climate change impacts have depicted that earth’s surface temperature has been warming more rapidly since the beginning of 19th century (Böhm et al., 2010; Hansen et al., 2010; Rohde et al., 2013; Hegerl et al., 2019; Carton et al., 2021). Based on the temperature fluctuation records taken over earth’s surface, seas, and oceans, it has been observed that the global mean temperature has increased by 0.8–1.5°C since the beginning of 19th century (Solomon et al., 2007; IPCC, 2019), and most of this temperature rise was seen after 1975 (Pierrehumbert et al., 2000; Hansen et al., 2006). Climate change occurs from three major factors, one from natural factors, secondly from human-based actions like GHGs and methane (CH_4_) emissions, and thirdly from changes in land-use. Human-induced anthropogenic activities have increased the atmospheric carbon dioxide (CO_2_) levels from 284 to 410 ppm between the time period of 1832–2013 (Hoffman et al., 2014), respectively, which ultimately caused the rise in temperature due to global warming. It has been projected that different factors involved in climate change will cause a rise in temperature, changes in precipitation frequency and intensity patterns, and probably incidence of more severe extreme events such as droughts, floods, and heatwaves (Solomon et al., 2007; Schiermeier, 2018). Global warming and its associated changes have already brought various modifications to biodiversity and human systems on earth’s surface (Kotir, 2011). It is anticipated that global warming patterns will not be even across the globe where arid and oceanic regions will be threatened more due to consequent impacts of global warming and extreme events (Solomon et al., 2007; Spinoni et al., 2021). Meanwhile, recent climate changes reports have depicted that the earth’s surface temperature will increase more slowly than projected from climate models due to the absorption of CO_2_ by oceans (Balmaseda et al., 2013). Livelihoods of coastal areas will be under more threat of floods and salinity due to anticipated climate change-induced sea level rise. There are uncertainties regarding rainfall patterns, specifically in tropical regions, due to the inefficacy of climate models to present the hydrological cycle with more accuracy (Lorenz et al., 2012). Climate change growingly altering the precipitation patterns worldwide, both in terms of amount of precipitation, duration, and timing. Generally, it has been envisioned that the duration of summer monsoon in Asia will increase, whereas northern and southern regions of Africa will become comparatively drier which depicted abrupt shifts in mean and extreme precipitation patterns (Shongwe et al., 2009; Gebrechorkos et al., 2019).

Extreme weather events caused by climate warming, such as low-temperature stress usually in spring, has constituted an adverse challenge to food production (Barlow et al., 2015; Jackson et al., 2021). Low-temperature stress has posed serious threats especially to crop production across the globe (Crimp et al., 2016; Ferrante et al., 2021; Zhang et al., 2022). It has been estimated that in several parts of the world, yield reductions because of low-temperature stress of winter cereals often causes 100% production loss. Even under optimized management practices, low-temperature stress in spring can decrease the long-term average production by 10%, causing great economic losses (Chauhan and Ryan, 2020; Collins and Chenu, 2021). In Australia, Africa, and Asia, spring cereals especially wheat has suffered from frequent low-temperature stress events and reduced the grain yield to a great extent (Holman et al., 2011; Hassan et al., 2021). Low-temperature stress during spring depicts that the temperature in the season of rebirth, growth, and development rises rapidly, and during late spring season, the temperature is generally lower than optimum. Following this, when the crop development enters the anther differentiation phase, the resistance to frost will descend sharply (Zhang et al., 2015, 2019, 2021). This will subject toward fruitlessness or adverse production losses sometimes 30–50% once the crop plant encounters low-temperature stress (Zheng et al., 2016; Liu et al., 2019). Many previous studies have reported that low-temperature stress during critical growth stages in major cereals significantly reduces the photosynthetic rate of plants, causing accumulation of carbohydrates which leads toward altered hormonal contents and enzyme activities (Wang et al., 2017; Liu et al., 2019; Yan et al., 2022). For optimized plant metabolic and energy transformation processes, photosynthesis is the essential source, however, it is greatly sensitive to abiotic stresses (Shah et al., 2020b), such as drought and high- and low-temperature stresses (Jin et al., 2021; Zhang et al., 2022). Spring low-temperature stress and other abiotic stress of such heavy metals impose oxidative damages (Ahmad et al., 2021a) and majorly impact the growth and development processes of plants by affecting important morphological and physiological processes, and then dry-matter accumulation and distribution, which subjects toward reduction in crop production, quality, and ultimately food security (Nurhasanah Ritonga and Chen, 2020; Aazami et al., 2021; Ahmad et al., 2021b). Hence, improving the plant metabolism, antioxidants machinery, osmolyte metabolism, and modulation in physiochemical attributes is necessary against biotic and abiotic stresses which also include soil heavy metal stress (Shah et al., 2019; Li G. et al., 2021).

Additionally, the rapid increase in frequency and intensity of extreme weather disasters which include floods, droughts, and wildfires as a result of climate change devastatingly impact food security and livelihoods. This rapid increase in natural disasters demands for substantial disaster risk reduction management measures, potential policies and intensified approaches in building resilience to the damaging impacts of climate change which can ensure a sustainable and productive future. Relative to previous decades, the annual prevalence of natural disasters is now more than three times as a result of uncertain and irregular climate change (Sloggy et al., 2021). Comparative to agriculture, industry, commerce and tourism considered as a whole, on its own agriculture sector suffers from the incommensurate share of nearly 63% of impact from natural disasters, where the resource-poor, least developed, and low- and middle-income countries carrying the major threat of these scourges (Ali et al., 2017). Therefore, between 2008 and 2018, agriculture sector had to suffer with a great economic loss of USF 108 billion during 2008–2018 in terms of damaged or reduced crop and livestock productions (FAO, 2021a). Such damages can be specifically more disastrous to livelihoods of communities holding subsistence farming with limited resources. Over the analyzed time phase, Asia was the region mostly hard-hit by climate disasters, with overall economic losses nearly USD 49 billion, followed by Africa with USD 30 billion, and Latin America and Caribbean with USD 29 billion (FAO, 2021a). Among natural disasters, drought is considered as the greatest perp of agricultural production losses, followed by flood, storm, pest and disease, and wildfire. New pests and diseases in crops and livestock have also become an important disaster hindering food security. These biological disasters can cause great economic losses in near future regarding crop, agroforestry and livestock and ultimately jeopardizing food security. Empirically, the impacts of climate change and natural disasters on agriculture, livestock, fisheries, agroforestry, and the natural resources and environment sectors vary at different stages. Considering agriculture, the direct and positive impacts of natural disasters are actually quickly identifiable such as typhoons enhance the supply and availability of water for agriculture. Alongside, floods greatly improve the soil fertility because they deliver nutrients from the upland areas to lowlands regions (Israel and Briones, 2012). Along with other yet-to-be-identified variables, the impacts of typhoons and floods are considered as positive because, they assist an increase in agricultural production in the affected regions and guide to improve the food security. In contrast, typhoon, wind storm, flood and drought stresses have the potential to decrease farm productivities, afflict farm resources, damage farm infrastructure, and limit farming options (Atanga and Tankpa, 2021). Moreover, individually, winds storms, typhoons and floods can limit the physical farm movement, supply infrastructure, supply routes, and may cause deaths or injuries to labor involved in farming. Consequently, the direct and negative variables may further subject toward indirect and adverse negative impacts on agriculture and causing heavy economic losses. Generally, due to frequent occurrence of typhoons, wind storms, floods and droughts, the general cost of agricultural and agroforestry production increases, meanwhile agricultural production decreases, consequently food supply declines and food prices increase (Kumar et al., 2022). Typhoons, wind storms, floods, and droughts can greatly impact and reduce vegetative cover, can lead toward soil erosion, higher coastal tides and storm surges in sea sides, can result in high siltation and sedimentation. Moreover, these disasters can also subject toward accumulation of several wastes, water pollution and distorted land topography, reduction in precipitation, lowered soil fertility and increased saltwater ingress (Shrivastava and Kumar, 2015; Akhtar et al., 2021). All of these phenomena may indirectly reduce the viability of land and water resources, reduce the ecosystem services and imperil human health and safety due to the prevalence of extreme event-related issues. Considering all disasters together, the potential direct and indirect negative impacts on agriculture and agroforestry threaten food security more obviously in the areas already vulnerable to climate change and natural disasters.

Climate variability is linked with a rise in temperature and change in precipitation patterns which alters the association among crops, pests, and diseases; and also changes various trends like water availability, pollinating mechanisms, and fisheries. Meanwhile, there are some benefits of increased levels of CO_2_ in terms of potential increment in crop production at mid-high latitudes, however, the increased concentration is causing global warming subjecting toward extreme climatic disasters. Though there exist solid evidences about the impacts of climate change on nutrition and mortality, but inevitably there are uncertainties due to limited understanding regarding the extent of climate change impacts on food security components (Nelson et al., 2009; Springmann et al., 2016; Shoaib et al., 2021). Therefore, reviewing the mechanisms of impacts of climate change on all components of food security and livelihoods is necessary to alleviate the research gaps essential in fulfilling the regional food security goals.

### Intertwined Relationships Among Climate Change, Agricultural Farming and Food Security

**FIGURE 1:**
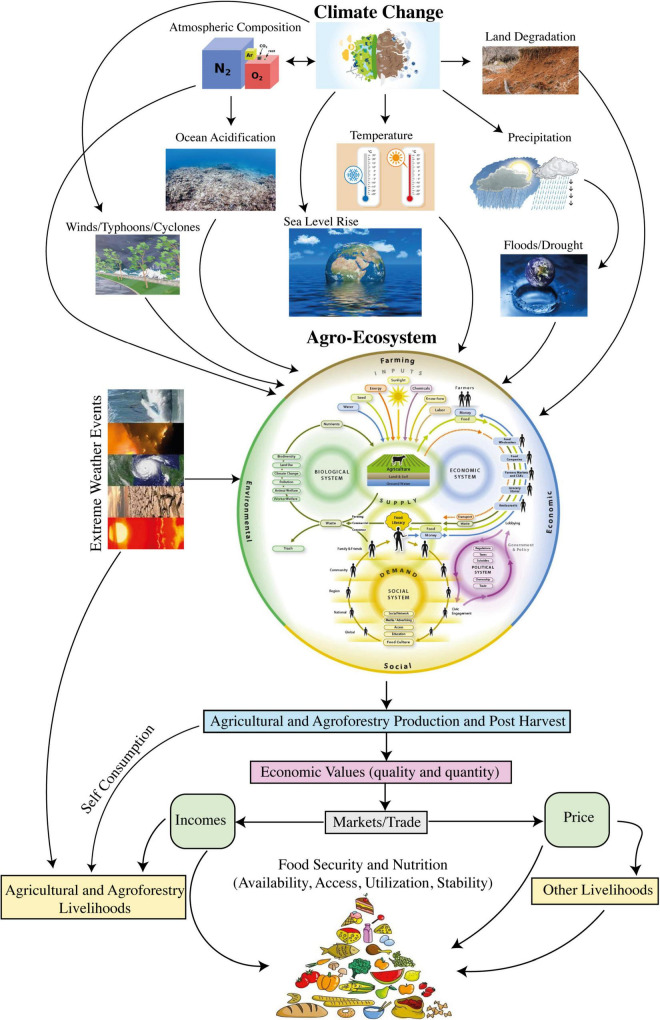
Schematic representation of climate change impacts on global food security and nutrition.

Climate change and extreme events will become key factors adversely affecting the food security and increasing undernourishment and malnutrition (Hughes, 2020). Considering the impacts of climate change, agriculture sector including crops and livestock is adversely affected and at risk due to subsequent vulnerabilities caused by climate change. Climate variability impacts agriculture and food productivity in various ways, exceedingly vary from global to regional levels (Tilman et al., 2011; Wu et al., 2014). Major food crops are impacted due to rise or fall in temperature, changes in rainfall patterns, global warming due to increased GHGs emissions, and soil abiotic stress caused by different heavy metal (Shah et al., 2020a; Raza et al., 2022) ultimately shifting the biological setups like crop cycle, insects, pests and diseases invasion, and growth periods. During 20th century, a longer crop life cycle was noticed as the most widely observed biological fluctuation in response to global warming in Northern Hemisphere (Steltzer and Post, 2009; Livensperger et al., 2019). Wheat and rice are among the major food crops that have reacted negatively to global warming in the last three decades, though the yield responses have still been under consideration and satisfied grain yields have been observed in various regions (Raza et al., 2019). Food production (net of food utilized for biofuels) must increase by 70% up till 2050 under the projected mean risen temperature conditions of nearly 4°C to meet the food requirements for an additional 2.3 billion population (totally 9.6 billion; FAO, 2009; Molotoks et al., 2021). However, to meet this food requirements by end of this century will be more challenging due to uncontrolled and uneven climate changes. Figure 1 represents the schematic representation of potential climate change impacts on global food security and nutrients intake.

During recent decades, an abrupt increase in atmospheric temperature has been observed in most of the Asian countries. Frequent water stress events, water shortage, and unsustainable and irregular intensive agricultural practices may adversely impact the future food security (Hameed et al., 2020). Almost 37% of GHGs emissions in Asia are associated with unsustainable agricultural practices (Syed et al., 2022). The agricultural sector including livestock, fisheries, forest farming and crop production is mostly affected by climate change and extreme events (Awan and Yaseen, 2017). It has been projected that shifts in climatic components will change the cropping seasons, negatively impacting the forest farming and forest farming-based livelihoods (Chisale et al., 2021), and could potentially permanently extinguish the viability of several crop and forest species at regional scales. Climate change is growingly threatening the crop production system of major crops, and it has been projected that expected rise in temperature will severely decline the productivity of major staple food crops such as wheat and rice leading toward various constraints in addressing food insecurity challenges in protracted crises (Maxwell et al., 2012). Climate change brings shifts in the precipitation and temperature which differently disturb the duration of different growth phases of the crops and forest plants (Davidson, 2018).

The forest farming and forest-based industries and climate change are closely interlinked depicting a key role for the development of climate-smart agriculture in improving the farmers’ livelihoods and sustainable forest management (Nkumulwa and Pauline, 2021). Forest farming and its industry influence the global carbon cycle via the sequestration of atmospheric carbon in forests and is successively impacted by global climate change through its influences on the forest growth rates and climate-induced shifts in natural disturbances. Similar to other agricultural sectors, the impacts of climate change on forest farming and its industries are different depending on the extent of climate variability, geographical features, and forest species. Climate change similar to crop plants change the overall productivity of forests, changing resources management, economic approaches of adaptation, and subsequently forest product harvests globally, nationally, and regionally (Keenan, 2015). In regions where climate variabilities reduce the timber growth, smaller timber volumes will be produced for harvest both in existing forests and those rejuvenated in near future. Moreover, climate change impacts the forest farming and agriculture sector differently which could subject to land use shifts that could be considered as one potential adaptation approach to improve livelihoods. Considering a possible example, if regional climate change causes relatively increased agricultural productivity per unit area, some area possibly be converted from forest farming to agricultural purposes depicting shifts in land use (Gurgel et al., 2021). Such shifts in land use would change the availability of forest products to international and regional markets, altering the prices of forestry products and the economic livelihoods of both producer and consumer. Consumers, ultimately, would modify their practices of consumption between forest and non-forest products. Meanwhile, producers would also change both the types of forest management practices and the wood harvesting time, which depend on type of owners whether public or private. Therefore, reviewing the adaptation of forest farming to climate change is essential for advanced understanding of the climate change impacts on forests, and forest-based industries to tackle the livelihoods of communities associated with forest farming. In addition, prediction and evaluation of how climate change impacts would change over time is necessary and integration of this knowledge will be helpful into the forest management decisions and policy makings (Nunes et al., 2022). However, it demands multiple types of new practices, knowledge, and adaptation approaches for suitable forest management decisions. Sharing that integrated knowledge from multiple agricultural, climate change, forestry, and food security disciplines will be helpful in building a shared set up for understanding the future challenges of food security and facilitating improved decision and policy making in the face of severe climate change (Keenan, 2015).

After climate change, mitigating the hunger and undernourishment threats is another major challenge of 21st century which threatens the food security goals (Jha, 2018; Webb et al., 2018). Hunger is associated with multiple ranges of concerns, ranging from nutrient deficiencies due to shocks in the accessibility of food to persisting food shortages. There are several intertwined relationships between malnutrition and poverty which subject toward hunger due to limited supply and availability of food in terms of quantity and quality (Siddiqui et al., 2020). Moreover, hunger is also caused because of the inability to buy sanitized and nutritious food, causing several infectious diseases leading toward poor health. Major hunger promoting agents have been addressed at wider scale in the last few years, which substantially encouraged the reduction in world’s undernourished population. Taking an example, due to suitable actions, the global undernourished population has reduced from 980 million to around 850 million between 1992 and 2012, respectively (Nawrotzki et al., 2014). Alongside, micronutrient deficiencies in routine diet have become a huge global health concern, impacting nearly one third global population. Micronutrient deficiencies render some important worldwide health issues, with malnutrition impacting major developmental outcomes like reduced physical and intellectual developments among children, vulnerability or aggravation of disease invasion, mental retardation, loss of hearing, blindness, and imprecise losses in body potential and productivity. Hence, it is necessary to comprehensively review the research gaps regarding dietary requirements and micronutrient deficiencies as 2 billion people are food insecure due to micronutrient deficiencies (Darnton-Hill, 2019).

It is put forward, based on the limitations and knowledge gaps, more studies on climate change impacts and consequences in terms of food losses are required, and the research focus should change to support implementation and capacity building to tackle food insecurity issues. Abundant information is available about recent advances in climate change and impacts on food production and food security, therefore, based on this, immediate actions are needed to tackle food insecurity challenges (Heal and Millner, 2014a; Schroeder and Smaldone, 2015; Campbell et al., 2016). In spite of having extensive progress in reducing the food insecurity challenges happened via enhanced food availability which is a major component in attaining food security goals, still the issues of undernourishment and nutrient deficiencies are stumbling. Globally, millions of people are suffering from micronutrients deficiencies, and problems of insufficient dietary intake. Therefore, bringing issues of hunger, malnutrition, and undernourishment into consideration is strictly needed to alleviate problems and challenges to meet global food security goals. It would be possible by reviewing the intertwined relationships among climate change, food production, poverty, malnutrition, undernourishment, and overall food security components. This study was designed to review such looped relationships between climate change and food security to reduce the research gaps hindering the food security aims. This article is reviewing three major mechanisms, firstly, the status of climate change and the challenges to food security responsible for increasing the research gaps. Secondly, how climate change negatively impacts the food security components and major research gaps. Thirdly, the possible potential options that how the challenges in climate change and food security research can be sorted out for better implementation in practice to alleviate the food insecurity risks.

## Flaws, Gaps, and Limitations in Climate Change and Food Security Research

### Lack of Interactive Research on Crops, Livestock, Forest Farming, Pests, and Diseases

Generally, previous studies on climate change impacts on food security mainly focused the agricultural crops and ignored livestock, forest farming, diseases, and pests. Moreover, the crop studies mainly focused on crop productivity with minimal consideration of value chains, landscape, and farming systems. Climate change also impacts the livestock farming which is an important component of food security (Godde et al., 2021). Livestock farming and its industry represents an important and key component of the agricultural economy especially among least developed and developing countries. Livestock farming contributes beyond direct food production which shares multipurpose provisions which include animal skins, fiber, fertilizers and fuels, and also capital accretion (Mahmood et al., 2014). In addition, livestock farming is are intimately associated with the social and cultural values of millions of resource-poor communities where livestock possession assures sustainable agricultural farming and economic stability. A significant increase in animal protein and fat usage has been seen in last few decades, which need to be increased up by 70–80% by 2050 (Herrero et al., 2015). It is strictly emphasized to deeply consider the climate change impacts on interactions of livestock farming and crops cultivation as the assessment of these interactions is critical for sustainable intensification, diversification, and climate change impacts management (Thornton and Herrero, 2015). Aquaculture and its industry already share a significant role in food and nutrition security across the globe. Yet, aiming to reach the full potential and providing sustainable and impartial aquatic food in the future, this sector essentially requires to innovate and anticipate projected challenges of climate change on food security. Globally about 1 billion people acquire their protein diet from fish, therefore, fish production has been spectacularly increased, where 41% comes from fish aquaculture farming (Beveridge et al., 2013). Likewise, forest farming and forest-based industry also play an indirect role in fulfilling the food security goals because they have a key contribution in the household food security, and economic sustainability of livelihoods as well as agricultural production systems (Cedamon et al., 2019). However, they could share a greater contribution to agriculture sector with more systematic and dynamic approaches to agroforestry with the identification and adaptation of innovative agroforestry measures in agricultural systems. Consideration of climate change impacts assessment both due to biotic and abiotic stresses inclusive of heavy metals on crop interactions and plant diseases prevalence (Ramzan et al., 2021) is also necessary as invasion of pests and diseases reduce global food production by 10–16%, which is critically more problematic in developing countries (Chakraborty and Newton, 2011; Elad and Pertot, 2014; Grace et al., 2015) and even complete crop loss if no countermeasures are undertaken. Alongside, outbreaks of emerging plant diseases and pests impact the food security, national security, livelihoods, and human health, with serious economic threats to agricultural economy (Ristaino et al., 2021). Several emerging plant diseases have already become more frequent due to climate change, and it is projected that invasion of diseases and pests will become more intense and frequent due to changes in their geographic distributions in face of climate change (Bebber et al., 2013; Bebber, 2015). Plant and livestock diseases influence all components of food security, and efficient management practices will subject to both improved food production, livelihoods, and human health. Most importantly, discussing the interactive impacts of climate change and emerging plant pests and diseases on food production and food security is necessary. Herein, it is necessary to evaluate the facts why plant pests and diseases emerge under climate change, and recommending an integrated research approach that can be implemented in prevention and control of pathogens, thereby improving adaptation and mitigation strategies to ensure food security. Therefore, it is needed to comprehensively review the interactions of climate change impacts, agricultural crop and forest farming, diseases, insects and pests under research considerations to tackle the food security challenges.

### Limited Considerations of Food Security Determinants

Generally, previous climate change and food security studies mainly focused on a single food security determinant, which quantified food mainly based on crop production ignoring other features. Future climate projections clarify that climate change will impact all components of food security, i.e., availability, access, utilization and stability, and ultimately the whole food system (Vermeulen et al., 2012; Noiret, 2016). Therefore, research focus should cover all components of food system (Porter et al., 2014; Campbell et al., 2016) rather only based on the analysis of climate change impacts on food systems solely on crops yield (Ziervogel and Ericksen, 2010; Sieber et al., 2015). Emerging and innovative studies should focus on the whole food system covering crop production, livestock and forest farming by understanding climate change impacts and implementing adaptation approaches in response to climate change. By this, problems on the demand side can be resolved to achieve food security aims under climate variabilities by taking action on the wastage of food and diets (Quak, 2018). Approaching the food system as a whole will benefit in delivering good nutrition to societies at a local scale rather than solely securing food availability on a global scale (Lang and Barling, 2013; El Bilali, 2019). The major determinants of food security vary at different scales starting from global branching toward regional and national to household and individual scale as food security is considered to be a holographic process surrounding climate change, civil constraints, climatic disasters, and socio-economic norms (Abdullah et al., 2019). Discussing all of the important factors involved in determinants of food security is essentially required to make the food system more sustainable. A number of factors are involved which influence the food security including household property (Tarasuk et al., 2019); economic constraints (Chang et al., 2014); education and awareness; livestock farming; cultivated land area; soil properties; access to market; resources availability; incomes; infrastructure; and awareness and knowledge for food production, storage, processing, and management. So, it has been revealed that gender, age, awareness, education, knowledge, remittances, employment constraints, inflation, possessions, and pathogens are some of the major factors influencing household food security (Abdullah et al., 2019). Hence, approaching the research-based evaluation of all above-mentioned factors influencing the food security and food system as a whole in face of climate change is necessary to sustainably cope with food insecurity challenges.

### Gaps Between Research Analysis and Implementation

There have been vast research-based consideration of climate change and food security, however, yet there are several research implementation limitations (Knight et al., 2008) which demands to review and navigate the space between research conduction and its implementation (Toomey et al., 2017). Climate change and extreme events impact both food security components and the livelihoods of those engaged in food production systems and their value chains. Moreover, climate change also affects the agricultural production systems, food supply chains and food pricing. In order to fulfill future rapidly growing population food needs, researchers must consider shifts not only in global climate change impacts and demographics on food security but also the degree to which food production systems can adapt against climate change (Burke and Lobell, 2010). Downstream, food access is associated with a stable and balanced food supply chain. Climate change impacts interrupt the food supply chain and disrupt the physical access to markets in various ways. Climate change-induced extreme events such as droughts, floods, and wind storms negatively impact the public infrastructure, damage market access facilities, inundate transport networks, and other health hazardous conditions for people to physically access markets (Nissen and Ulbrich, 2017). Research on a wider scale has been done for climate change scenarios development and impacts assessment, however, there is a lack of research considerations regarding adaptation measures against climate change and building adaptive capacity rather than merely forecasting the future climate. For example, IPCC 5th assessment report’s 1/4th part is focusing adaptation options, but in actual adaptation experiences were described on less than 1% portion, which apparently describes the research implementation gaps. There are various reports about the climate change impacts on crops, livestock, and forest farming but the scientific agenda to turn the analyses into actions against climate variabilities is still lacking (Herrero et al., 2015). Much analysis, but action paralysis is more noteworthy these days as on the other side of climate change impacts-adaptation options-action spectrum, there is minimal literature about adaptation measures, options and adaptive capacity improvement is available. Additionally, minimal work has been done so far about what works in different contexts, even if also considering current climate risk and vulnerabilities management options rather than measures needed for future climates (Campbell et al., 2016). Moreover, still there are several research gaps like just focusing on climate change impacts merely on food production, ignoring other components of food system and food security.

## Climate Change Impacts on Key Components of Food Security

Agricultural history is full of uncertainties and constraints where achieving higher food production and meeting food demands were based on increasing the cropping area and inputs along with applying new available technologies. However, traditional research methods completely ignored the interactive climate change impacts assessment on food security and food system as a whole which include livestock, fisheries and forest farming as well (Steenwerth et al., 2014). Agricultural crop production, fisheries, livestock, and forest farming in terms of quantity and quality depends on various kinds of physical and biophysical resources like soil health, availability and feasibility of natural inputs (water, sunlight, CO_2_, temperature), and sometimes pollination channels. Decline in food production commonly occurs due to climate change, natural disasters and also by pathogens and diseases. In some cases, food production and food availability are heavily influenced by availability of physical agricultural labor. Assessment of climate change impacts among different developing and underdeveloped countries where undernourished people are abundant showed an alarming and serious high hunger index among 53 countries. It has been observed that climate change has decreased the consumable calories in daily routine diet during the last few decades (Ray et al., 2019). It is concluded that climate change has boosted the issues regarding household food insecurity as it impacts all determinants food security and factors affecting food production, access, availability and stability (Godfray et al., 2010; Ziervogel and Ericksen, 2010; Mekonnen et al., 2021). Climate change influences all the components of food production and food security, but the impacts of climate change are essentially needed to be interactively characterized.

### Impacts on Food Availability

Food availability means if people have enough food to meet their dietary requirements and it also covers the supply side of the food chain. Food availability is determined through food production, technologies available, inventories, supply chain efficiency, and trade policies at national and international scales. Previous studies have vastly focused on the cumulative climate change impacts on cropping systems and food availability (Parry et al., 2005; Ali and Erenstein, 2017). Various studies focused on the impacts of future climate projections under various levels of CO_2_ concentrations, and that increased levels of CO_2_ likely to enhance crop productivity due to improvements in photosynthesis processes and improved water-use efficiency (WUE) because of high carbon fertilization (Lee et al., 2020). In contrast, it has also been found that crop productivity will increase with less rates in practical than projected in crop models (Long et al., 2006; Ainsworth and Ort, 2010). Therefore, the magnitude of change in crop production will vary branching from global to regional to country to community due to alternative and contrasting projections of crop models. Climate change impacts food production irregularly and unevenly as the impacts are more severe across tropical regions than in higher latitudes. Moreover, it has been projected those countries with a higher hunger index will be affected more severely in crop yield and livestock decline due to climate change. Forest farming and agroforestry contribute to household food and nutritional security in multitudinous ways. Trees directly provide a range of healthy foods such as fruits, leafy vegetables, seeds, nuts, and some edible oils. Forest farming can diversify routine diets and also address seasonal food and nutritional gaps. Forest farming also serves as a source of a wider range of edible plants, fungi, bushmeat, fish and insects. Forest farming and its industry also share a support in the provision of fodder for livestock, green fertilizer for crop production and wood-fuel crucial in many communities among least-developed countries for cooking food (Cedamon et al., 2019). Agroforestry and forest-based industry serve as a source of income in purchasing foods and also provide sustainable environmental services to support food production. However, there are various complexities in quantification of the relative benefits, profits and costs of forest farming-based systems in provision of routine food. These complexities in food provision quantification depict that the roles of agroforestry and forest farming-based systems are often not well considered and understood. Reviewing the research limitations and focusing the targeted research in forest-based systems can help in maximizing the farm benefits, productivity, enterprise, and sustainability (Monckton and Mendham, 2022). A comprehensive and deep understanding is necessary to focus on systematic ways to characterize the impacts of climate change on forest farming and ultimately food provision across different landscapes and on major indicators like dietary diversity approaches.

It is concluded that there is a vigorous and reasoned pattern of the climate change impacts on food production and food availability and it has been projected that uneven climate change will adversely impact the areas which are already under the prevalence of undernourishment and food insecurity issues. Systematic analysis of the changes in crop and livestock production across South Asia and Africa predicted that climate changes will decline the overall productivities of major food producing systems by 2050 (Knox et al., 2012; Sultan, 2012). The decline in crop productivities for major crops like wheat, maize, and sorghum will be vigorous, but the analysis was inconclusive and showed contradictive results for other major crops like rice and sugarcane (Knox et al., 2012, 2016). There are still some limitations regarding the broad impacts of climate change on food availability, though rational evidences for climate change impacts on crop productivities are available. Firstly, and more importantly, models that project and analyze the climate change impacts are only available for major cereal crops, few roots, and tubers. Climate change impacts some crops like pulses and vegetables which are considered as major income driven food crops locally (but globally minor) are deduced based on the same plant characteristics instead of solely analyzing them. Secondly, there are very few analyses about the grassland productivity and quality of livestock feed crops which curbs the understanding between climate change and livestock. Thirdly, most of the crop studies focus on the impacts of mean climate changes instead of capturing the weather extremes too, which sometimes can produce more adverse consequences on crop, livestock and forest productivities. Lastly, the understanding of climate change impacts on quantification of food provision and incomes from forest farming is limited which make food security more challenging.

### Impacts on Food Access (Measure of Affordability and Government Policies)

Accessibility to food can be defined in terms of the ability of a household to gain sufficient quality food to meet the dietary requirements (Ludi, 2009; Masipa, 2017). Acquiring a sufficient amount of quality food is only possible when households have adequate income resources for purchasing of food to maintain good nutrition levels (Fischer and Qaim, 2012; Fisher et al., 2012; Gartaula et al., 2017; Gupta et al., 2019). Food access is based largely on household income, capabilities, and rights. Food access issues are studied through two major pathways; firstly, from top-bottom models that address and link major fluctuations with household responses, and thereby the adaptation outcomes. Secondly, through community and household stages by trying to assess the climate variability impacts from bottom-up. Production of certain food products such as crops, fisheries, livestock and forest farming are transformed due to adverse climate change impacts at local, regional, and global levels which ultimately impact biomass production, including fiber, feed, food, or fuels. The shifts in land-use due to climate change impacts the overall food access by altering the geography of food system which involve crops, livestock and forest farming and impact the incomes at the farm level (Hertel et al., 2010; Zhao and Running, 2010; Hertel and Tyner, 2013; Peña-Lévano et al., 2019). Therefore, micro- and macro-level analyses for the climate change impacts assessment on every component of food access is necessary to help in meeting food security goals. Price of basic resources like water and land are based on long-term expected analysis, and most of the time, the prices border expected analysis of climate change such as the accessibility of water with a revaluation of land. Due to climate change impacts, some secondary structural consequences in a region or community may arise when there is a lack of property rights along with no protection for water and land resources which cause many food security problems, especially in developing and underdeveloped countries (Mendelsohn and Dinar, 2009; Godfray et al., 2010). These climate change induced structural consequences mainly target the poor people with abrasion of their resources of income.

Climate change projections and impacts on food access have shown that it will stress the people’s ability to purchase the food. Several IPCC assessment reports have demonstrated the negative impacts of climate change on the affordability of food through projections based on inter-linked climate, crops, livestock, forest farming and economic models. These inter-linked models project the prices of any agricultural and agroforestry food commodity and trade in coming future under several climate change, social, and economic scenarios, and projected results work-out the negative impacts on food purchasing power of any population under consideration (Nelson et al., 2014). There are several indications that show the micro- and macro-level climate change impacts on food affordability in the near future, and various climate change scenarios have demonstrated that climate food prices will increase depicting some uncertainties among the results projected through different macro- and micro-economic models (Nelson et al., 2014). Purchasing power of households impacts the affordability of food and ultimately food access which is considerably negatively impacted by climate change (Arnell, 2016; Tol, 2018). Most likely, climate change is expected to impact the geography of food production across the globe, like transference in suitable crop and livestock production regions that could cause considerable impacts on food prices, trade, and consequently food access (Havlík et al., 2014; Jones et al., 2017). Climate change also impacts the physical access of households to food by affecting the transport systems, road infrastructures and physical fortune (Tol, 2018). It is concluded that climate change adversely impacts the food access by affecting the food production systems and economic statuses.

### Impacts on Food Utilization

Food utilization to fulfill the dietary and nutritional requirements is strongly impacted by any mild change in climate along with other secondary factors like water availability and sanitation facilities. Very few studies are available to have broader consideration of climate change impacts on this component and determinant of food security. There is an obvious link between food utilization and climate change because climate variability limits the availability of food items such as drinking water (Kundzewicz et al., 2008; Delpla et al., 2009; Duran-Encalada et al., 2017). Undeveloped areas are more often devoid of sound sanitation systems, which cause hygiene issues during extreme weather events like floods or droughts (Hashizume et al., 2008; Seneviratne et al., 2012). Therefore, the non-availability of good hygiene systems causes different stomach diseases, which reduce the intake of essential nutrients, which is actually associated with temperature variations (Schmidhuber and Tubiello, 2007; Lloyd et al., 2011). Higher costs for foods are usually observed under climate change-induced events because climate change encroaches on diet quality; therefore, the demand for good hygiene food increased, which requires sound techniques to avoid food contamination from any adverse environmental factors (Paterson and Lima, 2010; Medina et al., 2014). Although there have occurred some advances in food utilization like food fortification and biofortification, however, there are still higher needs to bring innovations in food utilization and food science as a whole (Bouis, 2003; Nestel et al., 2006; Bouis and Welch, 2010; Bouis and Saltzman, 2017). Food security issues have been continuously increasing for many years, suggesting ways to strengthen the adaptation system under changing climate (Ziervogel and Ericksen, 2010; Molua, 2012). Improved adaptive capacities like improved income systems to expand the income from rich to poor, straightening employment programs for the poor, actions to fulfill childhood essential nutrients requirements, etc., need expansion to respond against climate change. A nutritional alteration will spread out in the coming decades due to climate variability, which requires the broader, potential and fortified considerations of these phenomena.

Food utilization is primarily influenced through two attributes: food safety dimensions through the food supply chain, and direct or indirect health impacts due to climate change that intermediate nutritional outcomes. Generally, high as well as low temperature stresses (Raza, 2020) are likely to reduce the food safety of necessary items especially fisheries, fruits and vegetables due to increased rates of microbial activities under changed environmental conditions (Marques et al., 2010a; Liu et al., 2013; Hammond et al., 2015; Galstyan et al., 2019; Alegbeleye et al., 2022). Climate change impacts food system in dynamic ways directly or indirectly such as vector-borne diseases, high and low temperature stresses, natural disasters which include drought and floods which consequently affect public’s income, nutrition, plus security and care provision to children (Connor et al., 2010). Human and livestock health is undermined and compromised food safety is observed due to water-related influences of climate change which include low sanitized water availability or increased contaminated water provision due to increased frequency and severity of drought and floods (Uyttendaele et al., 2015b; Djekic et al., 2016). Deep concerns have been expressed that new disease incidence will subject toward over and irregular use of pesticides for crops and agro-forestry and veterinary medicines for livestock and fisheries (Tirado et al., 2010; Sundström et al., 2014). Climate change indirectly impacts human health via loss of jobs and essential livelihoods, or migration and interposed public health services, which disproportionately influence indigenous communities and people who are already poor with negative consequences for food security (Ford, 2012; Hayes et al., 2018; Ebi and Hess, 2020).

### Impacts on Food Stability

Stability of food is greatly linked with changes in climate because climate determines the price trends for food, either long- or short-term variability in prices (Nelson et al., 2009; Myers et al., 2017). Since last decade, small blows in the food chain either at the demand or supply side, have impacted the prices, often increasing the food prices (Haile and Wossen, 2016). Food instability issues are more common among poor societies because poverty-stricken populations have to spend most of their incomes to buy high-priced staple foods. Climate change makes both the supply and demand sides of the food more unpredictable by increasing the food volatility (Gilbert and Morgan, 2010; Haile and Wossen, 2016). Climate change fluctuates the demand side, which puts food stability at risk; usually, it happens when political agendas and policies interfere, e.g., different kinds of subsidies either in crop and livestock or forest farming industry (Wheeler, 2015). These kinds of policy transitions have been implemented in various developed countries (United States, United Kingdom) due to energy considerations and climate mitigation and adaptation aims. A recent example of food instability showed that more or less food crises originated due to lack of short- and long-term adaptation and mitigation strategies against climate change and extreme events, less crop, livestock, fisheries and agroforestry productivities and lack of sound policies. The situation of food crises was aggravated by lack of policy implementation, less expertise due to restrictions from developed countries, limited access to lucid markets, and lack of price regulation systems (von Braun and Tadesse, 2012; Haile and Wossen, 2016; Headey and Martin, 2016). Therefore, concerns about food destabilization are continuously increasing due to climate change which is making more uncertain the poor populations’ food consumption through volatile food prices (Arndt et al., 2012a,b; Campbell et al., 2016; Roy and Haider, 2019). Some secondary risks with food destabilization arise more or less due to climate change, like economic and political risks that cause food insecurity issues for indigenous people (Berazneva and Lee, 2013). This complex aggregate of constraints and risks can potentially ride into a ruinous system for food security.

**FIGURE 2:**
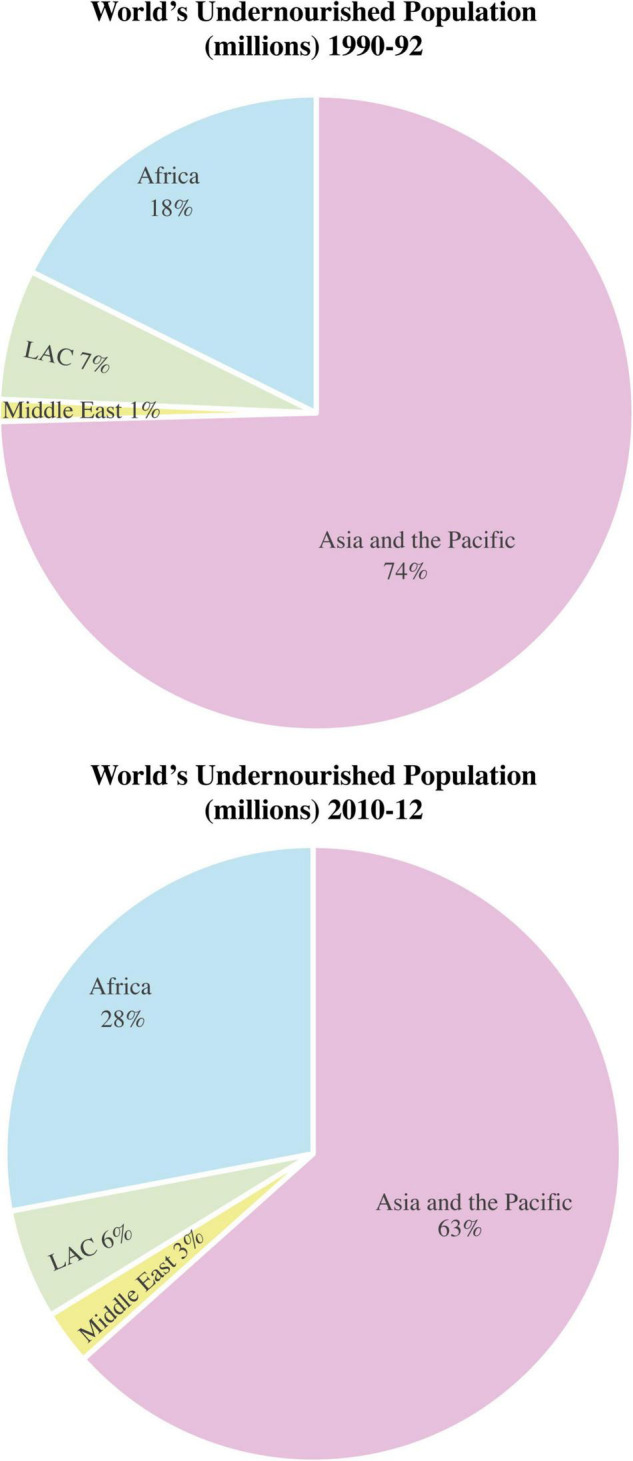
World’s undernourished population (The undernourished are those with a caloric intake less than the minimum daily requirement; LAC, Latin America and the Caribbean; Source: ADB calculations based on economy-level estimates from the FAO Food Security Indicators), http://www.fao.org/economic/ess/ess-fs/ess-fadata/en/.

There is direct and indirect linkage between food security and ecosystem which is based on the provision (food, water), regulation (climate, extreme events, pests, diseases), and support (water and nutrient recycling) services. The pressure on ecosystem is being intensified through climate change and extreme events (Berazneva and Lee, 2013). An increase or decrease in temperature and frequent prevalence of extreme events due to climate change bring in a decrease in biodiversity and also change the relationships among communities and within a respective community jumble (Mach et al., 2016; Oppenheimer et al., 2016). This ultimately put the agricultural and forestry productivities and food security on risk (Khoury et al., 2014). Climatic variability leads to various kinds of soil, water, livestock and crop related problems that consequently cause soil damages, shifts in soil properties, low surface and ground water quality, water availability issues, degraded food quality and quantity, and damages to human health and ultimately to ecosystem. Climate change, thus, also threatens the social and economic components of the food chain. Marginalized and resource-poor communities are easy targets for climate change by increasing their economic vulnerabilities and other socio-economic conflicts (Oppenheimer et al., 2016; Panpakdee and Limnirankul, 2018). Figure 2 represents the global variations in world undernourished population based on the calorie intake in the last two decades. At the start of this century, the world’s undernourished population was quite higher. Some developed countries took progressive steps but in spite of these steps, average world population of undernourished people increased in the 2nd decade of this century relative to the start of the century.

### Impacts on Overall Food Productivity

In spite of intrinsic shortcomings in climate-crop modeling, climate projections have indicated with some certainty that global food production will decline due to uneven and uncertain climatic variabilities (Porter et al., 2014; Martinich et al., 2017). Recent climate assessments with some uncertainties done by IPCC demonstrated that average productivity for major food crops (rice, maize, wheat) would decline by 3–10% with 1°C increase in temperature (Challinor et al., 2014, 2018). In the light of above findings, it has been observed that wheat production will decline by 6% with a 1°C increase in warming (Asseng et al., 2015, 2019; Hatfield and Dold, 2018). Besides, various findings have found that enhanced CO_2_ concentrations in air will make crops to produce more harvestable products especially in C_3_ plants (DaMatta et al., 2010; Ramalho et al., 2018). Climate change impacts on livestock production are conciliated by reducing feed qualities and quantities. Meanwhile, climate change will impact livestock and fisheries production through contention for natural resources, quality and quantity of feeds, livestock diseases, heat and cold stresses, and biodiversity loss. Moreover, the demand for fisheries and livestock products is projected to increase by 100% by mid of the current century (Rojas-Downing et al., 2017). Mysteries about how climate change impacts will occur and to what extent in the future are still under the unveiling process, and meanwhile the responses of forest farming toward climate change are even more uncertain. Some forest species are expected to become limited due to direct impacts of climate change which ultimately will hinder the economic growth and food security of populations whose incomes are concerned with forest farming. Forest productivity may be basically shifted if especially vulnerable, yet ecologically essential species are lost due to physiological impacts of climatic stresses (Kramer et al., 2020). Direct effects result when climatic variables approach physiological limits of forest and affect tree functioning. Identification of physiological limits of different forest species linked with livelihoods of different communities will help scientists describe their potential to survive in face of climate change. Climate change can also directly afflict the livestock food production by stressing the physiological processes. Taking an example of poultry and milk products which are considerably impacted in terms of quality and quantity when the temperature goes above the optimum range (≥30°C; Thornton and Gerber, 2010; Rojas-Downing et al., 2017).

Agriculture is the dominant source for fulfilling the dietary needs, but seafood also has the importance in the food chain to satisfy the protein, vitamins, minerals, and fatty acids for many societies across the globe (Bogard, 2015; Kawarazuka et al., 2017; Pradeepkiran, 2019). There would be a decline in potential fish production (5–10%) by 2050 specifically in tropical marine ecosystems (Barange et al., 2014; Lam et al., 2016). There are several projections about the expected shifts in fish distribution due to enhanced warming, fluctuations in nutrients availability, and changes in pH (Brander, 2010; Hollowed et al., 2013). Recent findings have shown that a decline in fish production will likely cause essential minerals and zinc deficiency in 845 million people, whereas B-12 vitamin and fatty acid deficiency in 1.4 billion people (FAO, 2016; Golden et al., 2016). A systematic analysis of nearly 5,000 fish farms worldwide reported that 68% of global fish production units have fallen below their potential biomass yield, which will lead to 88% loss of biomass by 2050 (Costello et al., 2016; Gaines et al., 2018; Bradley et al., 2019). Underdeveloped communities are at risk due to their limited resources to access dietary alternatives like fish, livestock, forest farming, and supplements. Additionally, there is a great association between climate change and the prevalence of pests and diseases. There are several risks associated with agricultural stability due to climate change as it will enhance the prevalence of various new pests and diseases (Bebber et al., 2013; Fones and Gurr, 2017). Table 1 represents the pathways of impacts of climate change on overall food production.

**TABLE 1:** Impact of climate change on overall food production (Thornton et al., 2009; Connolly-Boutin and Smit, 2016; Phiiri et al., 2016; Biglari et al., 2019).

Climatic components	Livestock	Food and feed crops	Labor and capital
Precipitation variation	1. Drinking water shortage 2. Pests and diseases	1. Reduced yield 2. Reduced food and food quality 3. Shifts in production systems	1. Shifts in human health 2. Migration 3. Reduced yield 4. Conflicts among and within a community
Temperature	1. Heat stress • Reduction in livestock productivity • Enhanced mortality 2. Pests and diseases • Reduced resistance against pests • Biodiversity loss	1. Reduced yield 2. Reduced food and food quality 3. Shifts in production systems	
CO_2_		1. Partial or total stomata closure 2. Shifts in production systems	

Insects, pests, and weeds are responsible for decreasing the productivity of major food crops, roughly 25–40%, according to several estimations (Ziska, 2004; Deutsch et al., 2018), despite the fact of limited global data. Fungal attack solely reduces the hygienic food availability by 8.5% across the globe (Fisher et al., 2012; Godfray et al., 2016; Udomkun et al., 2017).

**FIGURE 3:**
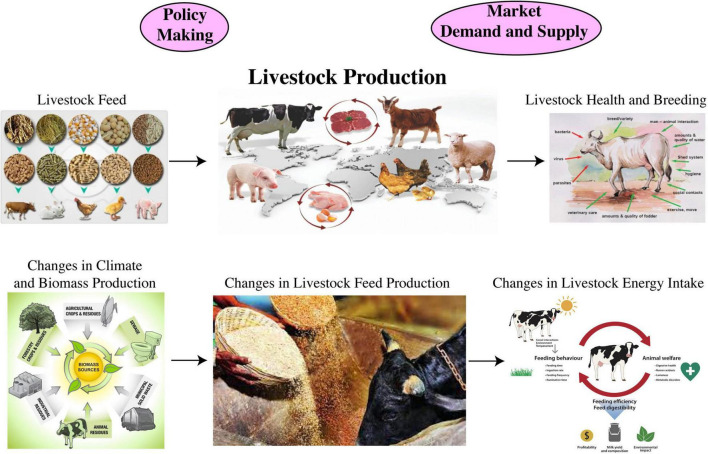
Impacts of climatic variabilities on livestock production including policy makings and market demand and supply.

Climate change, mainly the global warming increases the survival rates of insects, pests, and weeds (Bale et al., 2002; Chidawanyika et al., 2019), and temperature variation either toward cold or high ranges also bring some shifts in the latitudinal range of pests and diseases prevalence (Bebber et al., 2013; Deutsch et al., 2018). Indigenous crop varieties lack defense systems against non-native pests (Bebber, 2015; Bebber et al., 2019), which require possible breeding management techniques to cope with those new threats. Geographical mismatches between pests and pathogens may also subvert the biological control management (Donatelli et al., 2017). Climate change and extreme weather events undermine the agricultural production by providing recesses for better establishment of weeds, pests and diseases (Rosenzweig et al., 2001, 2014; Powell and Reinhard, 2015), however, sometimes extreme weather events increase the competitiveness of crops and livestock against pests (Seidel, 2014). Climate change is anticipated to enhance CO_2_ concentration, which leads to a shift in the composition of weeds and crop plant defense system against pests (Zvereva and Kozlov, 2006; Juroszek and Von Tiedemann, 2013; Myers et al., 2017). Elevated CO_2_ concentrations make the herbicide and pesticide less effective in controlling weeds and pests (Ziska and Goins, 2006; Varanasi et al., 2016; Ramesh et al., 2017). How food productivity on a global scale is impacted due to climate change is represented in Table 1, and the process of average potential impacts of climatic variability on overall livestock is shown in Figure 3.

### Impacts on Food Quality and Diversification

Climate change impacts food utilization, quality and diversification by influencing food safety mediating through the food supply chain, and nutritional challenges branching through climate change-induced health impacts. Ensuring sanitized utilization of quality and diversified will be more challenging as climate change likely to induce intense and frequent occurrence of extreme events which include drought, floods, cold stress, and heat stress (Raza et al., 2019; Haider et al., 2022). Meanwhile, in face of climate change and extreme events, the utilization of quality, sanitized and diversified food will be more challenging for resource-poor populations due to adverse impacts on livestock, crops, and forest (Marques et al., 2010b; Liu et al., 2013; Hammond et al., 2015). Moreover, climate change and extreme events limit the availability and utilization of sanitized food which subject toward several health risks (Costello et al., 2009; Wu et al., 2016). It has been projected that the occurrence of extreme events and natural disasters will be more frequent and intense posing various key threats to availability of diversified and sanitized food especially to resource-limited communities with more socio-economic conflicts. This would increase the health and nutritive constraints which in turn impacting the overall food security (McDonald et al., 2011; Uyttendaele et al., 2015a; Ziska et al., 2016; Hoekstra et al., 2018). Recently, frequent invasion of old and new pathogens and diseases in crop, livestock and forest farming systems have stimulated the enhanced use of pesticides and medicines which is also threatening the human-health (Tirado et al., 2010; Zhou and Turvey, 2015). Aiming toward getting higher food productions, irregular usage of different chemicals in livestock and fisheries, and overuse of synthetic fertilizers in cropping systems have put the human health at risk. Hence, it is concluded that climate change and intense occurrence of extreme events is expected to the livelihoods unevenly especially in developing and underdeveloped countries (Costello et al., 2009; Pillay and van den Bergh, 2016; Ford et al., 2018).

### Impacts on Overall Health and Nutritional Balance

Beyond the climate change impacts on crop productivity, fisheries, livestock, and forest farming, it also influences the nutritional configuration food. Enhanced CO_2_ levels in the air cause reduction in amino acid production and thereby protein contents in edible parts of crops and also impacting the nutritional value of vegetables (Dong et al., 2018). Comparative analyses among several cereal and legume crops showed the reduction in protein contents in former due to higher concentration of CO_2_ between 7 and 10%, whereas in later, the reduction was insignificant (Myers et al., 2014; Weigel, 2014; Dietterich et al., 2015). If these shifts in protein composition in plants continue, about 200 million people are likely to suffer from protein deficiencies, and among poor communities, it will get worsen and posing them at high health risks (Medek et al., 2017; Smith and Myers, 2019). Along with protein deficiencies, elevated CO_2_ also causes a reduction in essential minerals in major food crops. Taking an example from previous findings, if CO_2_ concentration encompasses 550 ppm, it reduces the zinc (Zn) and iron (Fe) levels by 3–11% in cereals and legumes. Moreover, if CO_2_ level reaches up to 690 ppm, it reduces the potassium (K), phosphorus (P), calcium (Ca), sulphur (S), and manganese (Mn) in a wide range of food crops (Loladze, 2014; Ebi and Loladze, 2019). More than 1 billion people across the globe are suffering from Zn deficiency, and if CO_2_ enrichment in air continues, it will bring-in another 200 million people under this deficiency (Myers et al., 2015; Beach et al., 2019). Overall, millions of people are expected to come under the risk of protein, Fe, and Zn deficiency due to increased CO_2_ levels, and the situation will deteriorate among societies already under the challenges of these deficiencies.

Temperature change stresses either cold or heat stress negatively impact the milk and meat production in livestock. Quality as well as quantity of livestock products is potentially and negatively influenced by temperature stresses (Bernabucci, 2019). Considering milk production, temperatures change especially heat stress has a more important impact on high-quality milk byproducts (Summer et al., 2019). Heat stress negatively impacts the organic and inorganic components of milk which lead toward strong associated changes in the byproducts industry of milk. These changes result in potential, negative economic outcomes to producers and consumers. Dairy cattle are comparatively more sensitive than beef cattle to temperature change especially heat stress, with their higher metabolic rate and higher body heat production. However, beef cattle compensate for increased body temperature by natural homeostatic mechanisms such as urination, panting, and sweating and various behavioral modifications which include decreased activities, enhanced water intake, and limited feed intake (Summer et al., 2019). Hence, it is concluded that at temperature changes especially higher than an animal’s thermoneutral range can significantly impact liveweight gain, milk and meat production, and also animal’s fertility (Thornton et al., 2022).

### Impacts on Food Nutritional Components

Access to food containing necessary nutritional components is dictated by political and economic forces. Prejudices based on gender, ethnicism, caste, and wealth hamper attaining the food security goals (Fortmann, 2010; Mearns and Norton, 2010). Climate change aggravates social ostracism through increased competition for diminishing natural resources, socio-economic factors and forced migration (Barnett and Adger, 2007; Hsiang and Burke, 2014). Moreover, climate change also brings in several public constraints that reduce the easy and full access to food that is necessary to fulfill nutritional requirements especially in South Asia, Africa and Middle East (Burke et al., 2009; Kelley et al., 2015; Buhaug, 2016; Levy et al., 2017). Historical data interpretation has shown that temperature change and inter- and intra-group socio-economic conflicts may arise in future decades due to limited fulfillment of nutritional requirements and will hardly hit the areas which are already facing the challenges of malnutrition and undernourishment (Hsiang and Meng, 2014; Buhaug, 2015; von Uexkull et al., 2016; Harari and La Ferrara, 2018). Therefore, such inter- and intra-group social conflicts may worsen the situation of undernutrition, malnutrition and ultimately food security.

Climate change and extreme events impact nutritional capacities of all kinds of communities because it aggravates social as well as economic pressure on the accessibility of quality food. According to the previous studies’ observations, inflation-adjusted prices for major food crops such as wheat, rice, and maize, will increase by 31–106%, considering the climate change mitigation and adaptation measures, rapid population increase, and income growth which will dictate the change in prices more appropriately (Nelson et al., 2018). The income and profit gains may preponderate the prices of expensive diets, and then laborers will get increased wages. Most analyses depicted that higher prices for foods will generally increase food insecurity issues not only for urban people for whom the impact is unequivocal but also for the poor people of rural areas where the majority of the people are net consumers (Ivanic and Martin, 2008; Martin and Ivanic, 2016). Recent analyses about food price versatility and food demand in under-developed countries depicted that higher food prices were linked with an increased reduction in food consumption among all communities, thereby concluding that higher food prices are likely to decline the consumption of the nutrients (Green et al., 2013; Herforth and Ahmed, 2015; Afshin et al., 2017). The overall shifts in the resources and food production are aggravated due to climate change which ultimately cause impacts on crops, fisheries, livestock and forest farming, thereby arising various kinds of nutrition issues under social, economic, and political conflicts.

Ensuring food security is branched beyond the demand and supply of markets. Attaining food security goals considers enough quality nutrition, which is only possible through protecting food against pests, diseases and spoilage. Necessary production and storage conditions will lead to have nutritious and healthy food to fulfill essential nutritional requirements (Hodges et al., 2011; Affognon et al., 2015). Lack of hygiene, poor production, storage and sanitation systems, and increased frequency and intensity of extreme events generally lead to more revelation to pathogens, insects and diseases, limiting essential nutrient intake, disrupting nutritional statuses, hindering normal growth, and development (Guerrant et al., 2013; Ngure et al., 2014; Gizaw and Worku, 2019). An ecological review among 70 countries worldwide between the time period of 1986–2007 showed that ease in access to good sanitation was greatly allied with reducing stunted growth among children (Fink et al., 2011; Fuller et al., 2015).

Future projected analysis for all components of food security (availability, access, utilization, and stability) will get disturbed due to fluctuations in mean change trends of crop, livestock, fisheries and agroforestry productivities, price fluctuations, income variability, pests, diseases and other socio-economic constraints. Meanwhile, lack of volatility, also usually named as stability, should also be considered in climate change projections. Due to climate change, food production patterns change spatially and temporally, and food prices may shift considerably limiting the food access only in reach of resource-rich communities. Yet, there are wider uncertainties in climate change projections and impacts on food system and determinants of food security. Hence, much focused work is required on the volatility of food access and utilization, although most of the economic, physical and biophysical models concluded that future world would experience more issues regarding food insecurity.

### Modern World’s Food Production Systems and Their Share in Climatic Variabilities

At the beginning of 19th century, the world’s population was just 1 billion, and three decades before, it has reached around 5 billion, and historical population increase data for last 20–25 years has shown that the global population has been increased by 2 billion (Van Bavel, 2013). With this rate of a growing population, it has been estimated that the global population will encompass 9.8 billion by 2050 and more than 11 billion by the end of 21st century where Africa will be main contributor (Islam and Karim, 2019). To meet this growing population food needs, it is necessarily needed to increase global food production multifariously. The competitiveness and increased food demand have transformed conventional agriculture toward modern agricultural systems through the use of synthetic inputs like increased use of fertilizers, and pesticides to get higher yields instead of following eco-friendly techniques (organic manuring, precision farming, fallowing, crop rotation). The agricultural sector is considered as one of the major sectors to contribute to GHG emissions, and due to increased deforestation and excessive use of synthetic inputs (pesticides, fertilizers), emissions in agriculture have been increased by 13.5% (Lenka et al., 2015; Bonou-zin et al., 2019). Embezzled and excessive use of synthetic N-fertilizers for higher productivity and to ensure food security consequently leads to increased nitrous oxide (N_2_O) emissions (Shcherbak et al., 2014; Griffis et al., 2017). Production process of synthetic N-fertilizers itself is the cause of many GHGs emissions (methane, N_2_O, CO, CO_2_) because of the burning of fossil fuels in the mechanized production processes. Thereby, increased emissions of N_2_O during the production of synthetic N-fertilizers and application cause a rise in global mean temperature due to its anthropogenic impacts (Wang et al., 2021). Therefore, frequent turn-out of extreme weather events is also caused by global warming led by irregular synthetic input use in agriculture. Water-cycles and quality also suffer from various shifts as synthetic N-fertilizers alter the water chemical properties when they are taken into lakes and rivers. So, water pollution is also being caused by synthetic N-fertilizers and excessive pesticide use in the agricultural sector.

The increased demand for healthy and nutritious food has subjected the small- and large landholders to engage more land area for perspectives other than agricultural and forest farming, leading toward an increased deforestation. Deforestation exacerbates climate change and frequency of extreme events as crop and forest farming is considered as the nature purifying components due to capacities to absorb CO_2_ and release oxygen (O_2_). So, deforestation appreciably stimulates the climatic variability and occurrence of natural disasters mainly through global warming, salinization, soil erosion, and desertification (Marengo, 2020). Fossil fuels are required to run the agricultural processes like production (pesticides and synthetic inorganic fertilizers), and processing (packaging, transportation, and distribution) which account for 1.2% share in agricultural GHG emissions (IPCC, 2015); however, this share does not include the emissions of agricultural inputs.

Preference-based food production also has a contribution to climatic variability. The luxury life-styles of upper- and middle-class populations and urban livelihoods have strongly changed the food preferences. Most of the nutrition intakes include meat which has increased by more than 350% in the last 50 years, and meanwhile livestock showed a major share in GHGs emissions (Sanchez-Sabate and Sabaté, 2019). Provision of a preferred meat diet has enhanced livestock farming which requires 20% more energy in production and processing than vegetables and cereals (Grossi et al., 2019). According to various estimates, livestock rearing for meat production emits huge amounts of CO_2_, which accounts for nearly 14% of all human-based GHGs emissions (Rojas-Downing et al., 2017). Based on the evidences, it is very clear that food production, processing and consumption have direct and indirect influences on climate variability. The enhanced emissions of anthropogenic gases speeds up the process of global warming ultimately leading toward natural disasters. Food production and processing influence food security as excessive use of synthetic agricultural inputs can damage the soil properties, and thereby reducing the ability of soil for crop or livestock production.

## Future Directions, Suggestions, and Challenges in Action Agenda

### Addressing the Climate Change Impacts and Achieving Food Security via Sustainable Development Goals

Now a days enough food is produced per unit area to feed the rapid growing population, however, yet nearly 811 million people are acutely suffering from undernourishment, amongst signals of decreasing impulse toward attaining zero hunger (FAO, 2021b). Meanwhile, malnutrition is sharing a massive toll across several developing and developed countries. Whilst, stunted growth, low height and decreased weight for age are gradually declining, where more than two billion adult individuals, teenagers and children are now obese or overweight (Ferreira et al., 2020). Hence, the consequences are becoming more adverse regarding public health, national economy, livelihoods, and quality of life. These adverse trends coexist with the abating availability of land resources, increased soil and biodiversity degradation, and more intense and frequent extreme weather events, where the impacts of these extreme events and climate change on agriculture amplify the situation. During a special meeting held on 25th of September 2015, the 193 members of the United Nations followed the different Sustainable Development Goals (SDGs; total 17) of the 2030 action-based Agenda for Sustainable Development among food systems, global objectives expected to assist the actions of the global communities over the next 15 years (during 2016–2030; FAO, 2021b). This 2030 Agenda of SGRs presents a fairer vision for more peaceful world where no one is left behind. All food systems including agriculture and agro-forestry are critical to achieve the intact set of SDGs where a special focus will be granted on rural development and action-based investments in different farming systems (crops, livestock, forestry, fisheries and aquaculture; Gregersen et al., 2020). This will empower the whole society with more powerful tools in alleviating the poverty, undernourishment, and hunger, and will lead toward more sustainable development. SGDs involve following 17 key points (FAO, 2021b) designed by commutative decision of United Nations (UN) Agency:

• Ending poverty in all its forms and everywhere
• Ending hunger, achieving food security goals, improving healthy nutrition, and promoting sustainable agriculture systems
• Ensuring healthy livelihoods and promoting well-being without any discrimination
• Ensuring inclusive and quality awareness and education for all communities while promoting long-term learning
• Achieving gender equality and empowering all especially women and girls
• Ensuring the availability of sanitized water with sustainable management measures
• Ensuring access to economic, affordable, reliable, sustainable and clean energy resources for all communities
• Ensuring and promoting inclusive, comprehensive and sustainable economic growth, employment opportunities and reliable working systems
• Building more resilient household and market infrastructures, transport systems and promoting sustainable industry development while fostering innovations
• Reducing inequalities within and among different nations
• Making cities more inclusive, safer, resilient to disasters and sustainable
• Ensuring sustainable food production and consumption patterns
• Taking urgent and necessary actions in combating climate change vulnerabilities
• Conservation and ensuring the sustainable use of the marine resources
• Sustainable management of forests, combating the desertification, hindering and reversing the land degradation process, and halting biodiversity losses
• Promoting justice, peace and inclusiveness among societies
• Ameliorating the global partnerships for sustainable development programs

However, the success of the SDGs reposes to a greater extent on an efficient monitoring, review and follow-up pathways as the foundation of this new global framework for mutual accountability (Glass and Newig, 2019). Moreover, healthy governance among institutions is necessary to achieve the SGRs through important approaches which involve participatory measures, reflexivity, policy coherence, mitigation, adaptation and most importantly the democratic institutions.

Action-based research is necessary to inscribe the climate change impacts on food security and the worldwide challenge to undermine the GHGs emissions from the agricultural sector. Past research findings have defined the dire need for both increased changes in agricultural production systems (like introducing new crop cultivars and better management measures for crops, livestock, fisheries and agro-forestry), and transformational changes (like trade shifts, diet changes, and motivating several environmental services; Hedenus et al., 2014; Newell et al., 2014; Baldos and Hertel, 2015; Hertel and Baldos, 2016). Immediate challenges inclined during coping with climatic variability and its impacts on global food security are described below;

### Challenges in Implementation and Suggestions for Action Based-Research

#### Incentivized and Motivated Research

**FIGURE 4:**
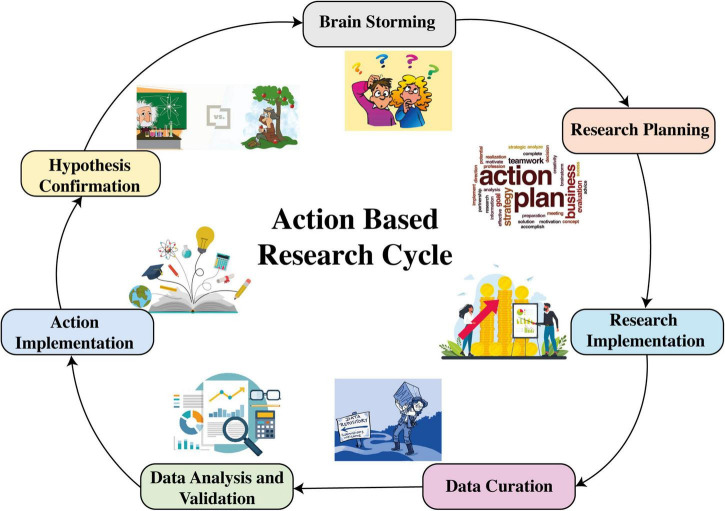
Important components of action-based research critical to ensure food security research gaps.

Food security and climate variability research are usually afflicted by uncertainties that require a focus on delivering-based research objectives involving local farmers and development agencies to share their experiences (Heal and Millner, 2014b; Naess et al., 2015; Campbell et al., 2016). Implementation of incentivized research and expansion of innovative research gains are usually very challenging (Figure 4). Therefore, narrowing the gap between research incentives regarding climate change impacts on food security and implementation is necessary. The expansion of uncertainties in climate change research makes the practical action less justified. Based on the experiences and findings of previous studies, it has been suggested to attain aims through action-based research, and dissemination of research experiences where focus of the research should be on the principle of “allocating the resources through needs, research, and capacity” (Fullana i Palmer et al., 2011; Vermeulen et al., 2013; Vermeulen and Campbell, 2015; Campbell et al., 2016). Following these, another principle should be undertaken based on tackling the powers and influences through understanding and engaging where stakeholders make usual decisions. Making efforts to make this principle stronger will make future research more optimized and focused for a better decision-making process among stakeholders (Naess et al., 2015; Döll and Romero-Lankao, 2017). Key mechanisms for better decisions, engagement of dialogs, and joint learning from experiences can only be possible if multi-stake holding platforms are ensured (Bhave et al., 2016; Ampaire et al., 2017; Huntington et al., 2017). Processes to develop climate scenarios need to be more struggled and accurate to empower the higher authorities and stakeholders for better policy making and implementation (Vervoort et al., 2014; Mason-D’Croz et al., 2016; Palazzo et al., 2017). No matter which kinds of processes or measures are taken, success measurement tools should show the nurture end results like policy making and its implementation. The incorporated tools and disciplines are customized following current and future challenges and opportunities. Although modeling regarding climate change plays an important role more often, but engagement process for stakeholders should be devoid of it to avoid more uncertainties. Evaluation of alternative climate scenarios, traversing the trade-offs, and clarifying hypotheses can be attained through modeling projections (Campbell et al., 2016; Little and Lin, 2017). Moreover, based on available uncertainties and knowledge gaps, interactive research including researchers and stakeholders should be ensured and must take realistic approaches to tackle current and future challenging climate change impacts through possible mitigation and adaptation solutions (Beven and Alcock, 2012; Beven, 2016; Krysanova et al., 2018).

#### Expansion of Possible Actions at Local, Regional, and National Levels

Uncertainties in climate change research and impacts of climate change on food security have made the research implementation process more challenging i.e., how the experiences could better be dispersed at three different scales, viz. local, regional, and national. Availability of natural resources is becoming scarce, and therefore, research should be more targeted with multiple potential benefits. The implementation of any research hypothesis should be driven with more care and short- to long-term prioritization (Bauer et al., 2015). Inscription of impacts of climate change on global and regional food security requires context-prioritized research conduction, actions and experiences sharing, and cross-dimensional and multi-level experiences implementations. Sector-based policy-making and action plans improve the collaborative work and implementation process in an optimized and efficient way. An effective approach for policy making and implementation intends to integrate the collaborative measures among different sectors involving food production, processing and storage which can be applied from local to national scale (Hallegatte, 2009; Nassopoulos et al., 2012; Orsato et al., 2017). For example, policy makers intend to bring some on-farm developments through technical interventions, while collaborative implementation must include supportive tools for wider development of technologies among different sectors.

Criteria for better implementation of hypothesized technologies should explore the tools having high anticipated outcomes, and collaborative impacts through linked local prioritizations and knowledge experiences. Thereby, adopting such feasible and potential tools make easier to address the current or near-future shifts, threats, and challenges (socioeconomic, economic, social, and political) associated with climate change impacts on food security (Notenbaert et al., 2017). Time of implementation and disbursement of specific research-based plans and experiences are very critical because some tools are necessary to be implemented immediately like climatic variability assessment. Whereas, remaining implementations can be can be shared later in the near future through projections of climate fluctuations and specific policy measures for capacity building. Collaborative priorities can be recognized through decision-support and policy implementation tools like wider-narrow climate modeling tools, local-national group scale planning measures, and such approaches can pliably be regulated at different levels accompanying multiple stances (Campbell et al., 2016; Challinor et al., 2018; Nikas et al., 2018). Potential mitigation and adaptation measures with optimized flexibilities are developed through collaborations between researchers and stakeholders’ operated processes. Because, actual and ascertained risks, uncertainties in research results, and interests may shift with the concerns and priorities of researchers and more obviously the stakeholders. Comprehensive approaches for focused research conduction and capacity-building are necessary for local as well as national scale benefits of any research experience sharing and implementation through local as well as national governments, respectively (Campbell et al., 2016; Chaudhury et al., 2017).

Moreover, long-term knowledge sharing through proper education and short-term training programs at local level may have key roles in affecting the design and implementation of any successful mitigation or adaptation measure. Short- and long-term trainings and experience sharing workshops may prove useful when customized to specific needs of attendees, are participatory in designing and implementation and tailored exploiting context-particular illustrations (England et al., 2018; Mataya et al., 2019). Action-based planning, on-the-job training, and extended leadership after experience sharing trainings can also be effective, but are rarely implied. Challenges that hinder improved capacity building associate not only to training and workshop design and structure, but also the exiguity of knowledge sharing assessments and the organizational structure. Stringent collaboration and monitoring of experience sharing efforts and befitting institutional assistance for action following sharing executions can be effective to improve mitigation and adaptation planning.

#### Ensuring Adaptation Measures Against Climate Vulnerabilities and Risks

**FIGURE 5:**
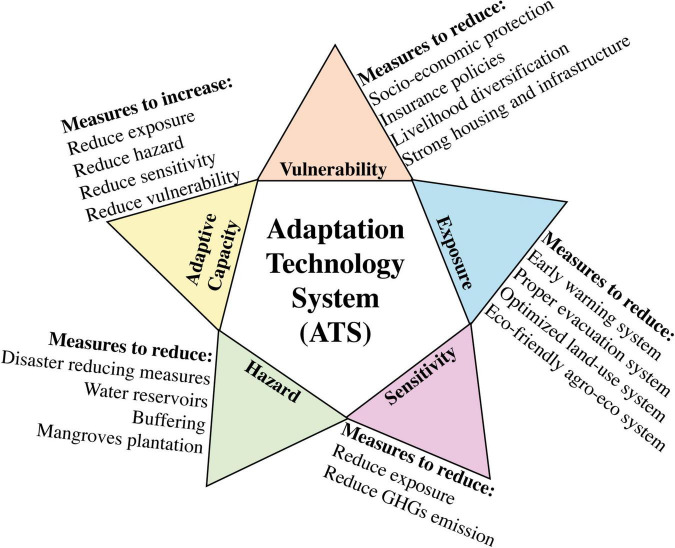
Important components of adaptation technology system (ATS) and adaptation measures for a resilient food system.

Ensuring adaptation measures against distinctive climatic vulnerabilities and risks is more challenging than the above-mentioned challenges (Figure 5). Climate change vulnerabilities include various geographical, social, economic, and political elements on which an individual’s or household’s resources are based to ensure food security under the geometry of climate variabilities (Sugden et al., 2014; Bellman et al., 2016). Gender-based differentiations impact households’ subjection to vulnerabilities and risks and provide tools to control resources, technologies, and services (Quisumbing et al., 2015; Johnson et al., 2016). Human health security especially women are usually negatively impacted in situations of declined household resources under climate change and natural disaster conditions like droughts and floods which impede the investments and on-farm activities (Sugden et al., 2014; Ani et al., 2022). Adaptation measures are affected under conditions where women are devoid of the necessary information and lack extended participation in social institutions under climate shocks especially in rural areas (Alam et al., 2017; Wood et al., 2017; Biesbroek et al., 2018). So, to undermine the inequalities and enhancing adaptation process, it is needed to ensure the optimized implementable actions at every scale branching from national to local. Gender-based suitable information resources and contexts to address the activities, attentiveness, literacy concerns, and access requirements are needed to make wider ingress against climatic variabilities. Moreover, gender-receptive and farmer-guided technological upheavals are essential to design improved and sustainable food systems (Waters-Bayer et al., 2015; Lemessa et al., 2019). Sustainable food systems with improved mitigation and adaptation measures are inevitable to reduce the gender and other socio-economic constraints and enhance multiplicity, which can uplift food production to fulfill household’s nutritional necessities.

#### Joint Mitigation and Adaptation Approaches Ascertaining Food Security

**FIGURE 6:**
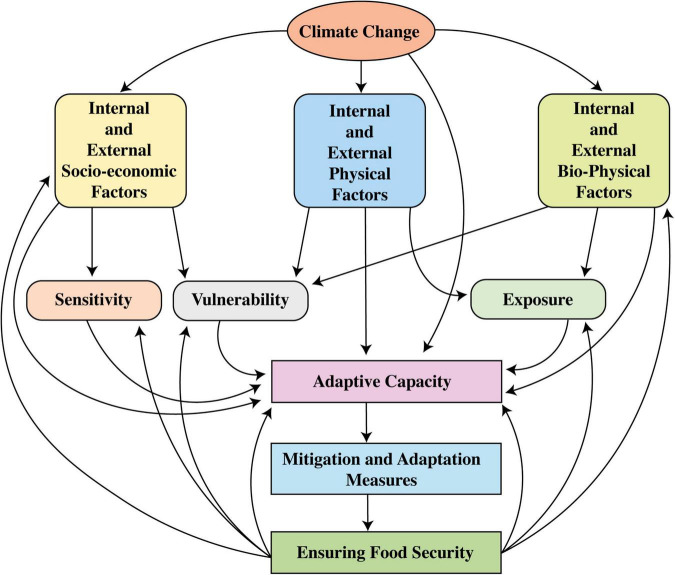
Adaptation and mitigation measures to ensure food security.

Food production should increase by 50% by 2050 to meet dietary requirements of rapidly increasing population (Alexandratos and Bruinsma, 2012; Hunter et al., 2017), but this increase will boost GHGs emissions, especially in regions with low productivity rates. So far, to reduce global warming trend by 2°C by the end of this century, IPCC climate scenarios have depicted that agricultural and human-induced GHGs emissions must be reduced to a greater extent. To meet projected food security goals for increased population through minimized share in climate change, sustainable food production systems need to be developed for low GHGs emissions to avoid environmental pollution. Therefore, it is very challenging to identify measures and approaches to fully secure food security goals under low GHGs emission pathways (Figure 6) as human-activities to ensure food demands and other livelihoods increase share to climate change (Myers et al., 2015; Golden et al., 2016, 2017; Smith et al., 2016; Smith and Myers, 2018). If such measures and approaches are identified in food system and food supply chain, communities can easily tackle joint challenges regarding mitigation and adaptation.

Sustainable agricultural approaches like confiscating carbon release and reducing future carbon emissions are promising to meet food security aims. Sustainable agro-forestry measures increase production, enhance use-efficiency for inputs, reduce food losses and ensure environmental safety through reduction in carbon emissions. Future food productivity can be gauged using approaches to reduce GHGs emissions relative to food production for identification whether a relative increase in production reduces the GHGs emissions (Murray and Baker, 2011; Cavigelli et al., 2012). Mitigation and adaptation approaches in face of climate change should narrow down the gap of GHGs emissions through increased crop and livestock production and reduce GHGs emissions to secure food security under sustainable food production systems (Sylvester, 2019). Many technological adjustments are available for incremental change, including GHGs emissions in cropping systems and livestock farming, and undermining the emissions from excessive fertilizer inputs. Many of the management practices in agricultural systems have already proved promising and sustainable in terms of food production without compromising environmental safety. For example, alternate wetting and drying system for irrigation and eco-efficient fertilizer managements (green manuring, organic manures) can be adopted as agronomic adaptations to reduce water and nutrient losses, thereby increasing resource use-efficiency with increased yield. Aerobic rice can be an eco-efficient transformation to traditional rice system (Farooq et al., 2022) to sustain the rice production with increased input use-efficiencies under future projected climate change and scarcity of resources (Fukai and Mitchell, 2022).

Concrete affirmations are required to make agree the investors and government agencies about the approaches to decrease GHGs emissions in agricultural systems with increased net production. Robust information is required about financial practicability and investments needed to blow-up approaches that also include costs and benefits for local farmer communities to replace their conventional approaches with new ones with good maintenance ability. Progressive agricultural production systems, agroforestry, different agro-ecological zones, and different geographic regions should be targeted where the implementation of practices to reduce GHGs emissions is feasible without negative impacts on the sustainability of the respective systems (Altieri et al., 2015). This implementation requires comprehensive scenarios involving shifts in climatic variable, food production, food demand and land-use. The positive and negative impacts of the preferred practices on already vulnerable communities would require to be foreseen and tracked to gain comprehensive future development goals.

Substitutional innovative modifications in agricultural and agroforestry systems are required to create more promising conditions where the levels GHGs emissions can greatly be reduced. Such approaches may include breeding measures aiming to reduce methane emissions in paddy fields, large-scale crop breeding and agronomic management measures to incorporate nitrification inhibitors in cereals, and transformational techniques in livestock to reduce the waste of food and organic manures. Policy measures, motivational approaches, knowledge and experience sharing and implementational optimized targets should be specified to encourage politicized actions and investments to extend the adaptation and mitigation under climate change to ensure food security at all levels. Developed and developing countries are now sharing several common adaptation priorities considering agricultural sector under more threat, protecting water reservoirs and resources, concerning toward climate change impacts on health, investigating the risks pose to the energy sectors, undertaking the safety of local livelihoods, and efforts to reduce the climate change risks (Hossain et al., 2017). Several underdeveloped countries have arranged several climate changes bills, national climate change strategy and national climate change action plans which identify the state’s priorities toward climate change risk reduction (Mall et al., 2019). Some developing countries including South Asian states have constructed climate change trust fund, and climate change resilience fund to invest in actions across riversides, environmental protection and disasters management (Dicker et al., 2021). Some developing states have established national councils for environment and sustainable development. However, these states still have limited progress at sub-levels and lack the capacity development for management. Several underdeveloped countries with aid of internationally developed nations have updated their medium-term development plans branching from national to grassroot district and municipalities levels because local climate change adaptive facilities assist adaptation strategies on pilot basis (Hertzler, 2007; Buurman and Babovic, 2016). However, insufficient and limited institutional capacities development, economic budget constraints, and inadequacy in attention to the potential of plans to develop climate resilience have impeded the potential merits of climate change adaptive policy plans in developing and underdeveloped countries. There can be several critical takeaways;

• Particularly, the key differences among different countries cannot be attributed to the development statuses where the states with higher stages of capacity development, are less actively involved in adaptation planning and policy developments than underdeveloped countries. Likewise, adaptation engagements also cannot be attributed exclusively to the fluctuations in exposure to climate stresses and interpret vulnerabilities to climate variabilities. Instead, adaptation progress seems to be impelled primarily by peak government leadership, and impacted by the precedencies of development assistance agencies in every state (Sovacool et al., 2017).
• Across all of the major countries worldwide, agriculture has been identified as the first priority for adaptation against climate change where it has been the focus of the major proportion of adaptation programs during recent years. This is possibly very unsurprising presented the climate-sensitivity of food production from crops and livestock, different governments’ wish to fulfill the food security demands of their populations, and the proceeding importance of agriculture as a major source of employment in various developing states (Sitati et al., 2021).
• The capacity development of governments at sub-levels in identification, prioritization, mainstreaming and implementing adaptation programs seems to be very restricted. Primarily in states where decentralization is ongoing, strict efforts are required to improve the capacity of local governments, communities, and institutions to take on their assigned duties. Greater accent may be constructed on elucidating the roles and duties of governments at different levels and launching essential institutional managements (Williams et al., 2020).
• It is necessary to assess the efficacy of financial investments in adaptation and mitigation action plans by governments of developing and underdeveloped countries along with their assisting partners. Moreover, necessary monitoring programs are essential to audit the overall progress in implementation of adaptation policies, plans and programs. Significant financial investments are needed to constitute, handle and properly utilize the investigation, monitoring and evaluation systems (Ramoutar-Prieschl and Hachigonta, 2020).
• Inadequate attention has been given in meeting adaptation requirements among many developing and underdeveloped states in various sectors (agroforestry, health, fisheries, livestock) designated as being primarily more vulnerable to the climate change impacts. The reasons for this ongoing trend are uncertain; they could be due to fluctuating national government preferences internally and externally, changes in directions on immediate development requirements, or the lack of substantial international and national financing institutions for adaptation in food providing sectors (Aryal et al., 2020).

#### Role of Financial Inclusions in Ensuring Food Security

**FIGURE 7:**
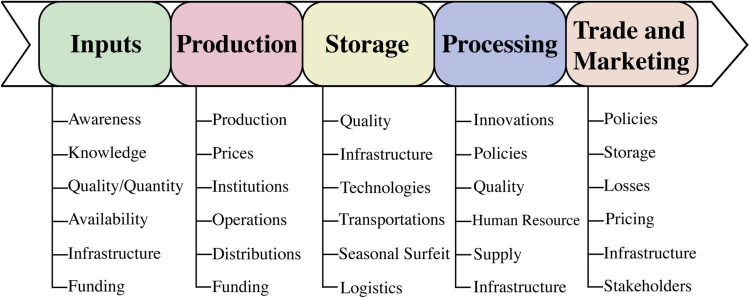
Role of financial services to address the issues of food security worldwide.

Agriculture is considered as the main direct or indirect source of employment and income for most of populations in developing countries, however, nearly 1.4 global population are world’s poor holding only 1.25 US dollars income a day (Rahim et al., 2014). According to an estimation, approximately 80% of the food utilized among developing nations by different farming systems of smallholder farmers (Ricciardi et al., 2018). But these farmers are generally vulnerable due to food insecurity challenges, and at the peak point of addressing the problem worldwide. Most of the smallholder farmers live in areas that are usually lacking easy access to necessary financial services, therefore, exposing them vulnerable to disasters and more prone to low-risk, low-return investments which subdue food productions and net incomes. It has been summarized that several supporting approaches including financial services at different scales can assist in ensuring and achieving various incentives of SDGs and promoting food security (Gil et al., 2019). Easy access to various kinds of financial services can support farmers get profitable investments that enhance their productions. Considering at a macro scale, higher food productions enhance the total food supply worldwide and directly add to the gross domestic production (GDP) of the resource-limited and poor communities predominantly in countries of Asia and Africa where economies are mainly based on agriculture. While at a micro scale, increased food yields contribute to enhance household income and achieving food security for nearly 1.5 billion global population striving in smallholder households (Rahut et al., 2022). The formal and informal financial services greatly share their role to reduce the risks and vulnerabilities to food systems in face of climate change (Figure 7).

Generally, financial services and products that are contrived for the particular demands of farmers are effective in efficiently increasing input access and elevating outputs. It is well known that farmers’ income generates all at once during food harvesting time, however, they need finances at other times of the season to acquire essential inputs and to smooth their utilization between food harvests. Sometimes the farmers are subjected to severe natural disasters that can demolish their farming outputs and expend their investments, and meanwhile several conventional financial services or even the financial products created for the urban poor communities may not suit farmers’ requirements (Chapagain and Raizada, 2017).

Different researches have focused to address the seasonal variation in smallholders’ income by providing easy access to state-oriented consumption and utilization loans through involvement of stakeholders at national and local scales during the hunger and stress seasons (Bonuedi et al., 2021). The cognition to smooth income empowered farmers to generate larger and, in few instances, to a greater extent profitable investments that boosted outputs and food utilization. An analysis conducted in Sub-Saharan Africa depicted key importance of input loans structured and designed specifically around the particular requirements of smallholders with terms and conditions to acquire a large lump sum amount during food production and repayment of the loan after harvest (Beaman et al., 2015; Fink et al., 2020). These collaborative loans oriented and supervised at state level involving public and private sectors can help in increase investments in farming inputs and subject toward an increased food production. African-based analysis on microcredit showed no measurable impacts on profits, however, it demonstrated the potential benefits of microcredit products and other microfinance services structured to address the seasonal credit requirements of farmers. This analysis depicted that pending the potency and efficiency of the loan-giving stakeholders such as agricultural banks, the measures like the inventory credit system are profitable and beneficial in assisting the credit access to smallholders (Dossou et al., 2020). Moreover, it is also necessary to combine the credit supplies with financial education, training, and technical support in a food production system for example the contract farming (Ncube, 2020).

Likewise, spreading weather-based insurance programs in low income and more vulnerable countries can also help the food producers invest in riskier, speculative and enhanced profitable input investments, which ultimately again subjecting toward an increased food production (Sibiko et al., 2018; Hirsch, 2020). In fact, it has been observed that insurance programs were even more effectual than credit to increase input investments (Carter et al., 2018; Chemeris et al., 2022). However, take up of insurance services among developing and underdeveloped countries remains low, and the provision of credit is usually constrained as it is considered very risky by financial departments. During missing or thing market conditions awareness and provision of simple products like savings bank accounts can also subject growers to acquire bigger investments in farming (crops, livestock, fisheries, forestry) inputs. Moreover, savings bank accounts are also considered as a cost-effective service for financial departments to pull new customers. To mitigate the negative impacts of climate change, it is necessary to undertake green banking initiatives through public and private sectoral partnerships involving banks, stakeholders and policymakers, although the approach used so far fractionally varies between developed and developing nations (Park and Kim, 2020).

Providing smallholders with awareness and service to automatically deposit some portion of their harvest revenue in savings accounts with involvement of local banks have been very effective (Stage and Thangavelu, 2019). This service impacts were observed and found that farmers who were offered and availed the choice of directly depositing some of their harvest proceeds into the savings bank accounts increased input investments by 13% and production by 21% relative to other who constrained this option (Brune et al., 2016). Moreover, this service also had potential positive impacts on household spendings, which strongly ensure food security for the smallholders. Summarizing the whole discussion, it is concluded that well-structured financial inclusions clearly exhibit that empowering smallholder farmers through financial services can increase income, input investments, and food production, strongly subjecting toward an improved food security. In this regard, government policies, stakeholders, and collaboration of public and private sectors in necessary for improved food production which depict a scoping retrospect to aware researches and policies for healthy and nutritious food commodities (Lencucha et al., 2020). So far not all financial products help farmers in developing countries especially smallholders because of having limited access to conventional financial inclusions. One major reason is that most of the smallholders live in rural regions, and far-off from most of the formal financial service institutions (Fan and Rue, 2020). Another explanation is that many financial services do not consider the seasonal investment and revenue opportunities of farmers or the sources of risk they confront, so farmers may not be entitled for or concerned in adopting the financial products, and they may not be beneficial even if they execute. Designing financial products around farmers’ particular financial requirements during any stress period can have potential impacts at micro as well as macro scales on improving food security for the poor communities of worldwide (Goodwin et al., 2022).

Agriculture farming (live is facing major challenge in fulfilling the global food security aims and is projected to face even severer challenges under future climate change. Table 2 describes the overall goal of how and where improved financing is necessary and can be utilized to attain the joint objectives of adaptation, mitigation, and development against climate change especially in developing and underdeveloped nations. Whereas, Table 3 describes which internation and national funding agencies are currently working worldwide. Yet, agriculture is considered as much under invested sector which has vast potential areas for investments appropriately to help the developing countries in maintaining sustainable food production under climate change. It has been summarized that mainstreaming adaptation and mitigation approaches into agricultural development strategies, encouraging capacity building approaches, and concerning multi-stakeholders’ requirements are main experiences for successful finance aiming sustainable food production and achieving food security under climate change. Joint financing by different national funding institutions, NGOs, donors, national, and international climate change funding agencies (World Bank, UNO, Green Climate Fund, USAID, USDA, EU Global Climate Change Alliance, etc.) can play their key role in reducing climate change impacts and ensuring food security.

**TABLE 2:** Potential and necessary areas for financing in adaptation and mitigation programs to reduce the risks to food security in face of climate change.

Financing in climate change mitigation and adaptation programs ensuring food security
**Financing in water conservation infrastructure** • Innovations in irrigation infrastructures • Different water-based projects across the country • Proper land-use (contouring, terracing, water storage mini dams) • Integrated land, and water drainage systems
**Financing in agricultural farming (fisheries, livestock, crops, agroforestry) and technology** • Financing research projects for better investigation of climate change impacts on food security components, vulnerabilities, and risks • International and local technology transfer through financing in extension services • Financing for development of stress tolerant crop, fisheries and livestock varieties • Financing in breeding programs, water saving technologies, encouraging organic farming • Financing for eco-efficient ecological and agricultural practices across the state
**Financing for capacity building** • Financing for trainings, workshops, awareness agendas, education, print and electronic media at local community scale • Financing for capacity building to better development and implementation of approaches to reduce the negative impacts of climate change on food security components • Financing for proper planning, implementation and management of capacities at local community level • Financing to improve food producer’s capacities by encouraging local association setups at local community scale
**Financing to reduce vulnerabilities and risks management in face of climate change** • Financing for disaster insurance programs • Financing for subsidized agricultural and agroforestry systems • Encouraging extreme events release and food help systems • Financing for technology systems to provide timely information and early warning systems, thereby for early management against weather predictions and projections • Financing to restore the natural capacities to buffer climate change impacts on food security determinants **Financing to reduce GHGs emissions from agricultural farming** • Financing for increasing fertilizer use efficiency • Financing for provision of trainings • Encouraging technology transfer • Financing for extension services **Financing for agricultural mitigation to reduce negative impacts of climate change** • Financing for increasing fertilizer use efficiency • Financing for provision of trainings • Encouraging technology transfer • Financing for extension services • Financing to educate for reducing GHGs emissions from livestock and cropping systems • Financing for energy saving technologies • Financing for soil carbon sequestration

**TABLE 3:** Different international and national funding agencies in different continents currently working to address the issues of climate change and risk management.

Major policy sectors	Organizations and institutions
Development Banking	Asian Development Bank, African Development Bank, European Bank for Reconstruction and Development, Inter-American Development Bank, World Bank Group
Regional Cooperation	African Union, East African Community, European Union, American States Organization, Pacific Islands Forum, South Asian Association for Regional Cooperation
Development and Policy	Organization for Economic Cooperation and Development, South African Development Community, Asian Development Community, United Nations Children Emergency Fund, United Nations Development Program
Agriculture and Food	Food and Agriculture Organization, West Africa Rice Development Authority, World Food Program, International Fund for Agricultural Development
Human Migration	International Organization for Migration, United Nations Commission for Refugees
Security and Peace	United Nations Security Council, Organization for Security, Defense, and Cooperation in Europe, North Atlantic Treaty Organization
Marketing and Trade	World Trade Organization, Economic Community for West African States
Health Security	United Nations Populations Fund, World Health Organization
Risk Management	United Nations Office for the Coordination of Humanitarian Affairs, United Nations Office for Disaster Risk Reduction

#### Proactive Involvement of Stakeholders to Solve the Issues of Food Insecurity

**FIGURE 8:**
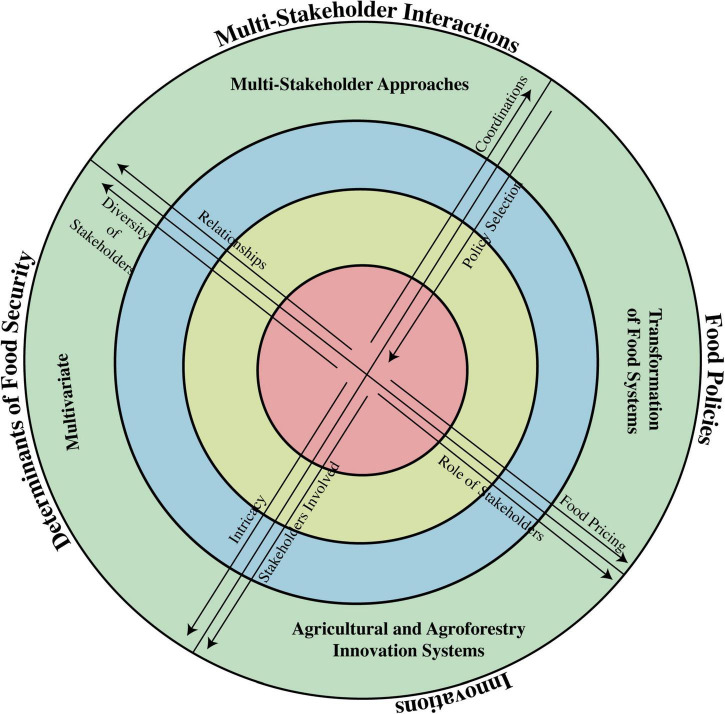
Importance of interlinked multi-stakeholder interactions to better address the food security challenges.

Multi-stakeholder engagements are indispensable in the development of public benefiting policies looking to encourage innovations in the face of multidimensional and complex challenges of climate change impacts on food security (Saint Ville et al., 2017). Globally, the ministry of agriculture primarily comprises two kinds of stakeholders, firstly the “policy members” which involve planning division, administrators, technocrats; secondly, the “extension members” having incentives to disperse the policies effectively. Policy members are given the tasks to efficiently manage the approaches to integrate all stakeholders in the planning, investigation, implementation, and evaluation processes (Saviolidis et al., 2020). Therefore, they play a key role in assisting the multi-consultative participatory processes. Moreover, multi-stakeholder engagement is necessary to ensure food security in areas contending with complex farming systems, and food insecurity issues. It has been observed that while being utilizing the multi-stakeholder approaches in developing nations, the stakeholder participations were restricted due to several factors with comprehended negative impacts on a particular policy coordination, integration and acceptance by stakeholder (Gaihre et al., 2019). There is a great importance of multi-stakeholder interactions in the design of policy development and implementation to improve food security. Figure 8 illustrates how policy developments and implementations drift away from narrow single dimensional measures toward multi-stakeholders’ coordination.

Informal and formal stakeholder interaction in the food production systems is necessary to assist the process of policy development and implementation at wider scale (Mohammadi et al., 2022). These interactions involve the function to serve as a think tank to advise the group on emerging challenges, proposing measures to optimized interventions, and proper monitoring of the policy framework. Farmer certification programs, provision of interest free loans to particular farmers likely to be more exposed to shocks and vulnerable, and relationships building between stakeholder and farmer are necessary for strikingness and positive impacts of stakeholder policies for food system and revenue generation. However, higher distrust level has been existing between smallholders and some stakeholder members that served to restrict the mutual knowledge streams required to support the development of food value chains (de Janvry et al., 2019). The major reason behind this is specific farmers usually trust their farmer colleagues rather anybody else holding an authority. Future approaches to adjudicate the complex challenges of food security in developing and underdeveloped countries, will likely in need of potent collaborations across different government ministries, improved rapprochement of policy constraints, and better policy innovations involving multi-stakeholder groups via the business of boundary organizations. Such approaches may have the capacity to reduce food security challenges via building more adaptive and pliant institutions, improving knowledge sharing and learning, and building trust among different stakeholders in the policy network (Breeman et al., 2015).

#### Role of Education and Awareness to Tackle the Issues of Climate Change Impacts on Food Security

Educating the locals about climate change impacts and awareness regarding impacts specifically on food security can assist in providing the active and dynamic knowledge about the values development; transformation approaches and acceptance; and imply knowledge generation, ultimately helping the local communities in creating and molding their basic strengths, abilities, knowledge, behaviors, skills and attitudes (Goldman et al., 2020; Smederevac-Lalic et al., 2020). Further, it helps the individuals in different social communities regarding the climate change challenges aiming toward managing better quality food systems and environment concerning food security (Rahman et al., 2014; Unsworth et al., 2016). In this regard, various studies have been conducted which provide theoretical and statistical indications, in characterizing the existing gap regarding actual statuses of pro-environmental behaviors, education about food security, environmental knowledge, adaptive capacity, and awareness (Geiger et al., 2019; Li X. et al., 2021; Ferreira et al., 2022). Similarly, adaptation approaches in face of climate change to ensure food security include variations in managing agricultural practices (fisheries, livestock, crops, agroforestry; Mumtaz et al., 2019; Aslany and Brincat, 2021). However, implementation of adaptation approaches is a complex, multi-dimensional, and multi–scale process, and most appropriately defined as shifts to strengths, behavior and economic structures so as to distill sensitivities of different communities in face of vulnerabilities and scarcity caused by environmental change.

Moreover, knowledge about adaptation approaches linked to food systems might include awareness about livestock breeding and diversified managements of crops; managing the land-use measures; shifts in the strength of food production which thereby involve shifting the land fragmentation (in case of livestock production) and the crop allocation; soil conservation practices; farm intensification and improved irrigation practices; and innovations in the techniques of farming processes (Chen et al., 2018). But there are certain challenges which squeeze the climate change awareness, knowledge about impacts on food security determinants and adaptation approaches vary from nation to nation and region to region (Abbasi and Nawaz, 2020). The issues and challenges about the education, and awareness to undertake adaptation approaches in face of climate change aiming ensuring food security are multidimensional, and recent studies have concentrated on several limitations to the climate change awareness and the adjustments besides apparent climatic and non-climatic agents of climate vulnerability, mainly in the developing and underdeveloped countries. Several studies have recommended that in face of climate variabilities, experiences are induced by both climatic (drought, temperature, floods) and non-climatic elements (limited availability of agricultural equipment and technologies, and reduced income) and therefore, it turns to be highly difficult to apprehend the intermix of such variables that aggravate the vulnerabilities of households against climate change (Jamshidi et al., 2019; Ashraf et al., 2021; Shah et al., 2021).

Region- and location-specific along with need-based information systems for farmers are necessary which will help in improving adaptive capacities by assisting decision making at basic level. Meanwhile, it is important to empower the locality-wise farming communities so that they evolve appropriate mechanisms for short- to long-term adaptation measures to reduce the uncertainties, vulnerabilities and risks caused by climate change (Asrat and Simane, 2018; Hansen et al., 2019; Stringer et al., 2020). Information, knowledge and awareness about climate change impacts assessment on food security must be regarded as one of the major media themes and be integrated as a matter of regular deliberated discussions in print and electronic media. Farmers must be provided with easy access to credible, applicable and well-timed information through advanced information and communication technology tools multi-collaborations of concerned government departments and stakeholders. Alongside, grassroot level campaigns, conferences, training workshops, awareness seminars, electronic media talk shows, timely declaration of stressful days and incorporation of climate change impacts on food security in conventional education curriculum are few strategies meriting government tendencies (Abbasi and Nawaz, 2020). Lastly, interdepartmental collaborations are crucial to hasten the implementation approaches, improve knowledge-based proper decision-making, encourage the climate and food security research and construct climate policies with strong scientific basis (Saina et al., 2013).

## Conclusion

The first three parts of this review focused on the climatic variability and its direct and indirect impacts on determinant of food security across the globe. There are still fundamental uncertainties related to the type and extent of climate variability, the responses of crop plants, animals, forests, human beings; and optimized adaptation measures in face of that irregular and uncertain variabilities. Although these uncertainties estimate exact future food production changes, however, it is clearly evidenced to be prepared for a wider range of potential negative consequences. Moreover, this review evidences that climatic variability is generally inclined against the environments which are already under climate fluctuations and have limited resources for mitigation and adaptation. Most of the cases represent that more focused and highlighted research priorities will reduce the uncertainties regarding future climatic projections. Describing the regional food priorities at a community level is very important, for example, estimation of food availability to know about the nutrition and health impacts branching from temperature change like cold stress or global warming and elevated CO_2_ levels. But this estimation only provides the evidences about the food availability rather than predicting actual access and intake of food across income differentiated communities and inadequately describes the food distribution at national and sub-levels. Additionally, there is a lack of global literature regarding the nutrient composition of a respective food and, if available, only restricted to several food databases that have not been rationalized for many years. Globally, we are facing an understanding and knowledge gap about people food access and intake for future that the dietary requirements for essential nutrients will be fulfilled, how and to what extent the climatic variabilities will impact crop, livestock, fisheries and forest farming at regional scales, and which health issues may arise in the near future.

Climate change, food production, and food security are all interconnected and entwined. Shifts in one bring direct or indirect negative influences on others. For example, a fast-growing population raises food insecurity issues; tackling such issues leads to a rise in food production through agricultural managemental measures (deforestation, extensive use of fertilizers and pesticides), which ultimately worsens the climate change process. Moreover, energy usage in crop production, fisheries, livestock, forest farming, and food processing processes accelerates the climatic variability. Frequent and intense occurrence of natural disasters due to climate change and impacts on food system (crops, livestock, fisheries, forest farming) and food security determinants need deep interactive research-based analysis for future where changes in one may cause adverse and diverse impacts on the remaining. It is very important to have deep insights into all of the determinants of food security and multi-sectoral impacts that influence the overall food security in face of climate change which are necessary to widen the adaptation and mitigation processes and ensuring food security. Moreover, an enormous range of factors (political, economic, and social) along with climate change and extreme events that share in food insecurity issues should also be considered to alleviate the research gaps of climate change impacts on food production (livestock, crops, fisheries and forest farming), availability, access, utilization, and stability.

Limited-researched areas and gaps include climate change impacts on wide cropping system challenges (such as value chains, value addition, crops in landscape contexts), on livestock and fisheries farming systems, on pathogens and new diseases, and on food security determinants other than merely on production. Disregard of uncertainties in projections of climate impacts to food systems and limitations in crop and climate modeling, it is evidenced that climate change impacts on food security will be more adverse, and therefore we advocate and urge for more focused research that will directly share the actions required to harness food security issues. Meanwhile, food systems will need some transformations in future decades, however, there are few immediate challenges. Firstly, to modify the culture of research basically to concentrate on outputs, where extensive engagement of multi-sectors will be required. Secondly, to design, contrive and trial different portfolios of alternatives where solutions will be extremely context-specific, therefore, it is needed to focus on highly prioritization approaches for the welfare of all kinds of communities locally and nationally. But this will also require extensive stakeholder engagement for targeted benefits. Thirdly, to achieve socio-economic comprehensions via an emphasis on people who are already more vulnerable to climate change and extreme events. Lastly, to address and consideration of mitigation and adaptation approaches together regarding food security, at local, regional, national and global scales. To meet food security in face of these challenges, climate and food sciences must work in collaboration with professionals, stakeholders, and policy-makers, to formulate key options in fulfilling current and future requirements and capacities, most importantly learning from gained experiences.

### Future Directions

In spite of flouring research work over the last decade, there remained impacts of climate change on food system and food security unknown. Having a better understanding of past and future climate change projections and evidences on overall food security determinants will be helpful to a great extent in achieving future food security goals. Strict consideration during research about climate change impacts on food security in terms of political, social, economic, and scientific consequences will ultimately reduce the uncertainties and be helpful in implementation of research experiences. However, some basic uncertainties will always interrupt because they are synchronized with climate change projections, climatic variability with time, and GHG emissions’ role in climatic variability. To overcome the uncertainties in climatic research, there is a strict need to focus on basic challenges regarding climate research. Firstly, it is very necessary to have an integrated understanding of food security; there is a need to find out the assemblage of authentications about climatic variability and its impacts on all components of food security. Secondly, there is a need to approach and model the exhaustive impacts of climate change on food security in terms of political, social, and economic consequences. Thirdly, it is required to have in depths future climate change projections and impacts on food security from global to regional to national to local, thereby, it would be easier to have sound climatic adaptations in food systems. Lastly, food system dimensions are totally dependent on the human behavioral reactions to actual and discerned climatic variabilities; therefore, it is requisite to have the blended human behavioral responses regarding climate change impacts on food utilization, availability, access, and stability. Addressing the above-mentioned four challenges will help in achieving the food security goals with a better understanding of climate change, food security, undernourishment and hunger.

Conclusive policy measures and action agenda is the need of time to reduce the food insecurity issues. There exists a large production gap between theoretical and practical crop, livestock and agroforestry productivities among many regions in this modern and innovative world (Lobell et al., 2009; Mueller et al., 2012; Chapagain and Good, 2015). Green revolution measures (adopting new crop cultivars, optimizing the use of inputs, developed irrigational systems) brought agricultural developments to many countries (Branca et al., 2011; Pingali, 2012; Aryal et al., 2018), but these development gains are limited to a few parts of the world. The unequal distribution of development measures brings critical food insecurity problems through declined food productivities (food insecurity in sub-Saharan Africa; de Graaff et al., 2011; Dawson et al., 2016). Critical food production gaps among various regions of the world require addressing the agronomic, social, political and economic constraints (Lobell et al., 2009; Reynolds et al., 2015; Myers et al., 2017). Agricultural innovations and climatic variability across the globe mutually determine future food productivity.

Measures to reduce food waste and loss will greatly help in meeting the food security goals. Primarily, unhygienic conditions cause fungal and other pests attack, which lead toward nearly 1.4 billion metric tons food loss every year, where most of the food loss exists in the developed world (FAO, 2014; Sheahan and Barrett, 2017). Although, rearing animals is an important nutritional and economic welfare tool for poor rural communities, however, crop production and agroforestry may directly help in easy availability of dietary energy (Cassidy et al., 2013; Shepon et al., 2016). Sustainable food production systems, better coping capacities against climatic variability like reducing GHGs emissions, and enhancing the input use-efficiencies for crop, agroforestry and livestock production can be helpful in alleviating negative impacts on food systems. Balancing the scope of policy priorities requires additional full understanding to account for how climate variability brings primary and secondary changes for food production and human health.

To implement the research-based findings practically, the decision stage needs to double-checked by decision- and policy-makers, because always challenged with anticipated climate change, impacts on food security though there are unlimited uncertainties among current findings and future projections. Moving toward practical implementations, there are few reconsiderations that decision-makers must undertake to concrete the alleviation process of climate change impacts on food security. Firstly, global decision-making organizations should prioritize those regions already at-risk regarding security food security due to severe climatic variability. Secondly, world’s silence seeing the global climate change impacts on food security in shape of hunger and malnutrition, which will potentially boost-up in the near future if kept ignored by developed countries. Thirdly, climate change potential impacts vary from global to a regional level, regional to local scale, and even vary among local communities, which raise issues regarding equitable food distribution at all levels. Therefore, projections of impacts at global, regional, national, and local scales and then to specify the practical implementations at a respective scale are important. Fourthly, poor groups and communities already at risk of climate variabilities are expected to be more vulnerable and prone to extreme weather events. Moving toward the fifth most important challenge, about the necessary adaptation measures to reduce the food insecurity issues in the near future that are expected to arise due to climatic variabilities happened in the last few decades as a result of bumper GHGs emissions. Lastly, periodic occurrences of extreme weather events will expand the uncertainties about the projected climate impacts, and global food security will be more vulnerable.

All the actual and perceived climate change impacts need comprehensive approaches to have sound mitigations and adaptations in reducing the global food insecurity issues. There are unlimited opportunities to develop sound adaptation approaches globally to undermine food security issues and to develop sustainable food production systems with better resilience against climate change. It is inevitable to modify the current agricultural systems toward climate-smart production systems through proper structural and functional adjustments to better cope with climate change impacts on all components of food security. This study encourages future research to deeply looking on different features of governance and their association with SDGs achievement, specifically focusing on each SDG goal individually. Studies assessing potential confined impacts of self-referent and adaptive governance approaches or policy coherence could impart to any attempt of SDGs. Being beyond the scope of the current article, a comprehensive assessment of the interactions between different dimensions and approaches of governance by using qualitative relative analyses would also share to further acquire sustainability in governance and its relevance for more specific SDGs implementation.

## Author Contributions

MF and MU conceived the idea, collected the relevant literature, and visualized the figures. MF, AR, MH, MY, MR, and MU helped to wrote the original draft. MF, MU, AR, YX, MR, and SY proofread and edited the final version. All authors carefully read, revise, and approved the article for submission.

## Conflict of Interest

The authors declare that the research was conducted in the absence of any commercial or financial relationships that could be construed as a potential conflict of interest.

## Publisher’s Note

All claims expressed in this article are solely those of the authors and do not necessarily represent those of their affiliated organizations, or those of the publisher, the editors and the reviewers. Any product that may be evaluated in this article, or claim that may be made by its manufacturer, is not guaranteed or endorsed by the publisher.
